# Association of pre-existing maternal cardiovascular diseases with neurodevelopmental disorders in offspring: a cohort study in Sweden and British Columbia, Canada

**DOI:** 10.1093/ije/dyad184

**Published:** 2023-12-27

**Authors:** Muhammad Zakir Hossin, Lorena Fernández de la Cruz, Kyla A McKay, Tim F Oberlander, Anna Sandström, Neda Razaz

**Affiliations:** Clinical Epidemiology Division, Department of Medicine Solna, Karolinska Institutet, Stockholm, Sweden; Centre for Psychiatry Research, Department of Clinical Neuroscience, Karolinska Institutet & Stockholm Health Care Services, Stockholm, Sweden; Department of Clinical Neuroscience, Karolinska Institutet, Stockholm, Sweden; Department of Pediatrics, University of British Columbia, Vancouver, BC, Canada; Clinical Epidemiology Division, Department of Medicine Solna, Karolinska Institutet, Stockholm, Sweden; Department of Women’s Health, Division of Obstetrics, Karolinska University Hospital, Stockholm, Sweden; Clinical Epidemiology Division, Department of Medicine Solna, Karolinska Institutet, Stockholm, Sweden

**Keywords:** Maternal cardiovascular disease, paternal cardiovascular disease, attention-deficit/hyperactivity disorder, autism spectrum disorder, intellectual disability, negative control exposure

## Abstract

**Background:**

We aimed to investigate the associations of pre-existing maternal cardiovascular disease (CVD) with attention-deficit/hyperactivity disorder (ADHD), autism spectrum disorder (ASD) and intellectual disability (ID) in offspring.

**Methods:**

This population-based cohort study included singletons live-born without major malformations in Sweden (*n *= 2 699 675) and British Columbia (BC), Canada (*n *= 887 582) during 1990–2019, with follow-up from age 1 year until the outcome, death, emigration or December 2020, whichever came first. The primary exposure was defined as a composite CVD diagnosed prior to conception: cerebrovascular disease, arrhythmia, heart failure, valvular and congenital heart diseases. The incidences of ADHD, ASD and ID, comparing offspring of mothers with versus without CVD, were calculated as adjusted hazard ratios (aHRs). These results were compared with models using paternal CVD as negative control exposure.

**Results:**

Compared with offspring of mothers without CVD, offspring of mothers with CVD had 1.15-fold higher aHRs of ADHD [95% confidence interval (CI): 1.10–1.20] and ASD (95% CI 1.07–1.22). No association was found between maternal CVD and ID. Stratification by maternal CVD subtypes showed increased hazards of ADHD for maternal heart failure (HR 1.31, 95% CI 1.02–1.61), cerebrovascular disease (HR 1.20, 95% CI 1.08–1.32), congenital heart disease (HR 1.18, 95% CI 1.08–1.27), arrhythmia (HR 1.13, 95% CI 1.08–1.19) and valvular heart disease (HR 1.12, 95% CI 1.00–1.24). Increased hazards of ASD were observed for maternal cerebrovascular disease (HR 1.25, 95% CI 1.04–1.46), congenital heart disease (HR 1.17, 95% CI 1.01–1.33) and arrythmia (HR 1.12, 95% CI 1.01–1.21). Paternal CVD did not show associations with ADHD, ASD or ID, except for cerebrovascular disease which showed associations with ADHD and ASD.

**Conclusions:**

In this large cohort study, pre-existing maternal CVD was associated with increased risk of ADHD and ASD in offspring.

Key Messages

- Epidemiological studies suggest that maternal cardiovascular disease (CVD) can alter placental function and lead to adverse obstetric and neonatal health outcomes.
- Most studies focused on short-term offspring health outcomes, and no study investigated whether the presence of maternal CVD before pregnancy increases later risk of neurodevelopmental disorders in offspring.
- In the current study, pre-existing maternal CVD (including cerebrovascular disease, arrhythmia, heart failure, valvular and congenital heart diseases) was associated with increased risk of attention-deficit/hyperactivity disorder (ADHD) and autism spectrum disorder (ASD) in offspring.
- Paternal CVD generally did not show elevated risk of ADHD, ASD or intellectual disability, warranting further research to identify the possible intrauterine mechanisms underlying the maternal-offspring associations.

## Introduction

Cardiovascular disease (CVD) is a major cause of pregnancy complications, morbidity and mortality in pregnant women.^1^ The high metabolic demands of the mother and the fetus during pregnancy require substantial physiological adaptations,^2^ which might not be well tolerated in pregnancies with a pre-existing cardiovascular history.

Whereas there is evidence suggesting that heart disease during pregnancy is associated with adverse maternal and neonatal health outcomes,^3^^,^^4^ the longer-term effects of chronic maternal CVDs on offspring, particularly referring to neurodevelopmental disorders (NDDs)^5^ such as attention-deficit/hyperactivity disorder (ADHD),^6^ autism spectrum disorder (ASD)^7^ and intellectual disability (ID), remain understudied. NDDs have a strong genetic component,^8^ and various non-genetic modifiable risk factors, including maternal health, may also contribute to their aetiology.^9^

Previous studies have documented elevated risks of NDDs in offspring of mothers with pre-pregnancy obesity,^10–13^ pre-gestational and gestational diabetes^11^^,^^14^ and hypertensive disorders during pregnancy.^15^^,^^16^ However, the potential association between pre-pregnancy maternal CVD and NDD outcomes in offspring has not been explored. From a causal inference perspective, a major challenge is to disentangle the effect of the intrauterine environment from genetics and other unmeasured environmental factors that might be linked to both maternal exposure and offspring outcomes. The paternal negative control exposure could be useful to address some of the unmeasured confounding factors.^17^^,^^18^

In this population-based study, we aimed to examine the association of maternal pre-existing CVD with ADHD, ASD and ID in offspring, using paternal CVD as a negative control. Since fathers cannot directly transmit the risk of NDDs through intrauterine environment, any possible association between paternal CVD and offspring NDD would imply the presence of residual confounding.

## Methods

### Data sources

The data were primarily derived from the Swedish Medical Birth Register^19^ and the British Columbia Vital Statistics Birth file^20^ which cover more than 98% of all births occurring in Sweden and British Columbia (BC), Canada, respectively. In Sweden, we cross-linked the Medical Birth Register to several national registers: the National Patient Register (inpatient and outpatient specialist care),^21^^,^^22^ the Cause of Death Register,^23^ the Prescribed Drug Register,^24^ the Total Population Register^25^ and the Education Register.^26^ In BC, the British Columbia Vital Statistics Birth database^20^ was linked to: the Discharge Abstract Database (hospitalizations),^27^ the Medical Services Plan physician billing data (outpatient physician visits),^28^ the PharmaNet (drug exposure)^29^ and the Central Demographics File^30^ (see Supplementary Table S1, available as Supplementary data at *IJE* online, for details of all registries).

### Study population

We identified 3 074 630 live singleton births with ≥22 completed gestational weeks from the Swedish Medical Birth register (January 1990–December 2019) and 1 176 936 live singletons from the BC Vital Statistics Births (January 1992–December 2019). Exclusions were made for infants with any major malformations [International Classification of Disease (ICD)-9 codes: 740–759; ICD-10 codes: Q00–Q99], missing national registration number for mother and children and children who died, emigrated or had the outcomes before their first birthday (start of follow-up). In the unexposed group, we excluded mothers with CVD diagnoses not part of our maternal CVD definition, except for hypertensive diseases (see Supplementary Table S2, available as Supplementary data at *IJE* online, for codes). Of the eligible children, 2 699 675 (92%) in Sweden and 827 896 (94%) in BC had complete data for analysis. For comparison, we linked 2 672 229 (99%) children in Sweden and 811 481 (91%) children in BC to their biological fathers with available CVD diagnoses data (Supplementary Figure S1, available as Supplementary data at *IJE* online).

### Exposure

Our primary exposure was maternal CVD diagnosed prior to conception (i.e. date of birth minus gestational age), including primary or secondary diagnoses of: cerebrovascular disease, arrhythmia, heart failure, valvular heart disease and congenital heart disease. We assessed both individual CVD subtypes and a composite maternal CVD. In Sweden, maternal CVDs were identified through ICD-9 or ICD-10 codes in the National Patient Register, which includes specialist care data (inpatient since 1987 and outpatient since 2001). In BC, the diagnoses were based on ICD codes in the Discharge Abstract Database or at least two records for CVD in the Medical Services Plan data between 1985 and 2019. Paternal CVD was defined similarly using the same ICD codes. The transition from ICD-9 to ICD-10 occurred in Sweden in 1997 and in BC in 2001. The validity of cardiovascular diagnoses in the Swedish inpatient register is generally high.^22^

### Outcomes

Offspring diagnoses of ADHD, ASD and ID were extracted from the first birthday until 31 December 2020 from the National Patient Register in Sweden and the Discharge Abstract Database and the Medical Services Plan in BC. ADHD cases were further identified through records of dispensation of ADHD medication using the Anatomical Therapeutic Chemical (ATC) codes retrieved from the Prescribed Drug Register in Sweden (since July 2005)^24^ and PharmaNet in BC (since 1996; Supplementary Table S1).^29^

### Covariates

The covariates selected a priori were: infant characteristics including sex, birth year, preterm birth (<37 gestational weeks) and small-for-gestational age (<10th percentile of the standardized birth weight distribution); maternal characteristics including mother’s age at delivery, parity, region of birth, marital status/cohabitation with partner, educational level (Sweden only), smoking during early pregnancy (Sweden only; self-reported at first prenatal visit or at 30 to 32 gestational weeks), pre-gestational diabetes, pre-gestational hypertension; and both parents’ history of any NDD or psychiatric disorders. Preterm birth and small-for-gestational age were considered as potential mediators, as they were previously shown to be associated with maternal heart disease^3^ and child NDDs.^9^ The registers from which specific covariates were obtained are shown in Supplementary Table S1 (available as Supplementary data at *IJE* online).

### Statistical analyses

We assessed neonatal and maternal characteristics in relation to maternal CVD status. Cox proportional hazard regression models were used to estimate the incidence rates and hazard ratios (HRs) with 95% confidence intervals (CIs) for overall maternal CVD and its subtypes. We evaluated the proportional hazard assumption using the Schoenfeld residuals method and found no strong evidence of violation for either the exposure or its subtypes. For each NDD outcome, follow-up began at age 1 year and continued until diagnosis of the outcome, death, emigration or 31 December 2020, whichever occurred first. We controlled for child’s sex and birth year in a minimally adjusted model, followed by a fully adjusted model that also controlled for maternal characteristics (including mother’s age at delivery, parity, education, region of birth, marital status/cohabitation with partner, smoking during early pregnancy, pre-gestational diabetes and pre-gestational hypertension) and parental history of any NDD or psychiatric disorders. To account for potential intra-cluster correlations among children of the same mothers, robust standard errors were estimated.

We performed a negative control analysis^17^ using paternal CVD status, to assess potential unmeasured confounding in the associations between maternal CVD and offspring outcomes. Paternal CVD serves as a negative control, sharing unmeasured genetic risk factors with maternal CVD but not including the hypothesized intrauterine pathway (Supplementary Figure S2, available as Supplementary data at *IJE* online).^17^ Since maternal characteristics might be correlated with paternal CVD through assortative mating and may affect offspring NDDs, we adjusted the paternal models for maternal risk factors.

All analyses were performed separately for Sweden and BC, with the fully adjusted HRs subsequently combined through fixed-effect meta-analysis using the inverse variance method. We additionally tested the cross-product interaction terms between overall maternal CVD and offspring sex with the use of a Wald chi-squared test, given the higher incidence of such outcomes in males compared with females. The BC data was analysed in SAS version 9.4. Stata version 17.0 was used to analyse the Swedish data and perform the meta-analysis.

### Causal mediation analysis

We undertook a sex-stratified counterfactual mediation analysis with preterm birth as a mediator (Supplementary Table S3, available as Supplementary data at *IJE* online). Since small-for-gestational age was not associated with the exposure in our initial investigation, we did not consider it as mediator in the final mediation analysis. The generalized linear Poisson model was used to decompose the total effect of maternal CVD on ADHD and ASD into natural indirect effect (i.e. the effect that goes through preterm birth) and natural direct effect (i.e. the effect that goes through other mechanisms). See Supplementary Methods S2 (available as Supplementary data at *IJE* online) for identification assumptions.

### Sensitivity analyses

We performed several sensitivity analyses. First, in the BC cohort where data on maternal education and smoking were unavailable, we evaluated their potential as confounders using the Swedish data. Moreover, because information on measured maternal weight in the Swedish Medical Birth Register was missing in 1990–91^19^, we included maternal body mass index (BMI; kg/m^2^) in an additional analysis. Second, to account for potential diagnostic changes over time, we repeated the main analysis, limiting the Swedish sample to children born from 1997 and the BC sample to children born from 2001 onwards, coinciding with the availability of ICD-10 codes. Third, in BC where aggregated data on average neighbourhood income were available (with 2.7% missing), we adjusted for parental neighbourhood income quintiles in maternal-offspring associations. Fourth, we also examined associations specifically related to offspring with ASD but without ID.^31^ Fifth, considering the possible influence of advanced paternal age on both paternal CVD and offspring NDD,^32^ we included paternal age as an additional confounder in the paternal-offspring associations.

### Missing data

Children with missing data on any study variables were excluded from the main analysis (Sweden 8.2%; BC 6.2%). See Supplementary Methods S3 (available as Supplementary data at *IJE* online) for missing data analysis.

## Results

In Sweden, among 2 699 675 children analysed (48.9% female), 22 775 (0.8%) had mothers with pre-existing CVD. Among these women with CVD, 13 064 (57%) had arrhythmia, 5534 (24%) had congenital heart disease, 3414 (15%) had cerebrovascular disease, 2069 (9%) had valvular heart disease and 515 (2%) had heart failure. In BC, out of 887 582 children analysed (49.1% female), 22 010 (2.5%) had mothers with any pre-existing CVD: 14 346 arrhythmia (65%), 4168 congenital heart disease (19%), 2111 cerebrovascular disease (10%), 2712 valvular heart disease (12%) and 718 heart failure (3%).

In both Sweden and BC, mothers with CVD were more likely to be older, multiparous, born in Sweden/BC, and live without a partner, compared with mothers without CVD (Table 1). They also had higher frequency of pre-gestational diabetes, hypertension and history of any neurodevelopmental or psychiatric disorder. Offspring of mothers with CVD were more likely to be born preterm.

**Table 1.** Maternal and offspring characteristics according to maternal pre-existing cardiovascular diseases: singleton offspring live-born without major malformations in Sweden 1990 to 2019 and in British Columbia, Canada, 1992 to 2019

Characteristic	Sweden			British Columbia, Canada		
	Total (*n*=2 699 675)	Maternal CVD		Total (*n*=887 582)	Maternal CVD	
		No (*n*=2 676 900)	Yes (*n*=22 775)		No (*n*=865 572)	Yes (*n*=22 010)
	% (*n*)	% (*n*)	% (*n*)	% (*n*)	% (*n*)	% (*n*)
**Offspring characteristics**						
Sex						
Male	51.2 (1 380 754)	51.1 (1 369 074)	51.3 (11 680)	50.9 (451 766)	50.9 (440 502)	51.2 (11 264)
Female	48.8 (1 318 921)	48.9 (1 307 826)	48.7 (11 095)	49.1 (435, 816)	49.1 (425 070)	48.8 (10 746)
Birth year						
<1995	18.1 (489 118)	18.2 (488 405)	3.1 (713)	12.2 (108 741)	12.5 (107 788)	4.3 (953)
1995–99	14.6 (395 250)	14.7 (394 033)	5.3 (1217)	18.0 (159 311)	18.1 (156 830)	11.3 (2481)
2000–04	14.9 (403061)	15.0 (400 913)	9.4 (2148)	16.8 (148 859)	16.8 (145 651)	14.6 (3208)
2005–09	16.5 (446 162)	16.5 (441 995)	18.3 (4167)	17.6 (155 860)	17.5 (151 776)	18.6 (4084)
2010–14	17.9 (483 033)	17.8 (476 523)	28.6 (6510)	17.8 (158 357)	17.7 (153 264)	23.1 (5093)
2015–19	17.9 (483051)	17.8 (475 031)	35.2 (8020)	17.6 (156 454)	17.4 (150 263)	28.1 (6191)
Preterm birth						
No (37–44 weeks)	95.7 (2 582 605)	95.7 (2 561 143)	94.2 (21462)	95.1 (844 077)	95.1 (823 450)	93.7 (20 627)
Yes (<37 weeks)	4.3 (117 070)	4.3 (115 757)	5.8 (1313)	4.9 (43 505)	4.9 (42 122)	6.3 (1383)
Small for gestational age						
No	93.7 (2 530 487)	93.7 (2 509 193)	93.5 (21 294)	97.0 (860 618)	97.0 (839 184)	97.4 (21 434)
Yes	6.3 (169 188)	6.3 (167 707)	6.5 (1481)	3.0 (26 964)	3.0 (26 388)	2.6 (576)
**Maternal characteristics**						
Maternal age at delivery (years)						
≤19	1.0 (233)	1.6 (43 648)	1.1 (195)	3.6 (31 689)	3.6 (31 129)	2.5 (560)
20–24	11.5 (2609)	14.6 (390 428)	12.3 (2177)	14.7 (130 718)	14.8 (128 080)	12.0 (2638)
25–29	29.9 (6819)	33.1 (886 069)	29.1 (5164)	29.1 (258 614)	29.2 (252 909)	25.9 (5705)
30–34	33.3 (7592)	32.5 (870 408)	33.3 (5912)	33.0 (292 339)	32.9 (284 778)	34.4 (7561)
≥35	24.3 (5522)	18.2 (486 347)	24.2 (4289)	19.6 (174 222)	19.5 (168 676)	25.2 (5546)
Parity						
1	42.9 (1 159 845)	43.0 (1 151 123)	38.3 (8722)	46.3 (411 422)	46.6 (402 965)	38.4 (8457)
2	37.1 (1 001 045)	37.0 (992 255)	38.6 (8790)	36.2 (321 296)	36.1 (312 816)	38.5 (8480)
3	14.0 (377 224)	14.0 (373 717)	15.4 (3507)	12.2 (108 347)	12.1 (104 920)	15.6 (3427)
≥4	6.0 (161 561)	6.0 (159 805)	7.7 (1756)	5.2 (46 517)	5.2 (44 871)	7.5 (1646)
Region of birth						
Sweden	79.8 (2 154 173)	79.7 (2 134 569)	86.1 (19 604)			
Other Nordic	2.0 (54 112)	2.0 (53 858)	1.1 (254)			
Non-Nordic	18.2 (491 390)	18.3 (488 473)	12.8 (2917)			
Region of birth^a^						
Canada				68.2 (605 499)	67.8 (587 095)	83.6 (18 404)
Asia				22.7 (201 376)	23.0 (199 166)	10.0 (2210)
Europe				5.2 (46 077)	5.2 (45 232)	3.8 (845)
Other				3.9 (34 630)	3.9 (34 079)	2.5 (551)
Cohabitation with partner						
Yes	94.3 (2 545 600)	94.3 (2 524 248)	93.7 (21 352)			
No	5.7 (154 075)	5.7 (152 652)	6.3 (1423)			
Marital status^a^						
Married				73.2 (649 457)	73.2 (634 042)	70.0 (15 415)
Divorced/separated/widowed				19.2 (170 526)	19.2 (165 922)	20.9 (4604)
Never				3.3 (29 316)	3.3 (28 392)	4.2 (924)
Other				4.3 (38 283)	4.3 (37 216)	4.9 (1067)
Level of education (years)^b^						
≤9	8.7 (233 481)	8.6 (231 537)	8.5 (1944)			
10–11	16.9 (456953)	17.0 (454 275)	11.8 (2678)			
12	24.1 (651 634)	24.1 (645 408)	27.3 (6226)			
13–14	15.1 (407 096)	15.1 (404 048)	13.4 (3048)			
≥15	35.2 (950 511)	35.2 (941 632)	39.0 (8879)			
Smoking in early pregnancy^b^						
No	88.7 (2 394 604)	88.7 (2 373 861)	91.1 (20 743)			
Yes	11.3 (305 071)	11.3 (303 039)	8.9 (2032)			
Any neurodevelopmental or psychiatric disorders						
None	92.9 (2 507 947)	93.0 (2 489 533)	80.9 (18 414)	91.2 (809 580)	91.5 (792 203)	79.0 (17 376)
Neurodevelopmental disorders	0.6 (16 465)	0.6 (15 960)	2.2 (505)	0.3 (3010)	0.3 (2796)	1.0 (214)
Psychiatric disorders	6.5 (175 251)	6.4 (171 398)	16.9 (3853)	8.5 (74 992)	8.2 (70 573)	20.0 (4420)
Pre-gestational hypertension						
No	99.3 (2 681 809)	99.4 (2 659 422)	98.3 (22 387)	93.4 (829 268)	99.5 (861 254)	99.3 (21 866)
Yes	0.7 (17866)	0.6 (17 478)	1.7 (388)	6.6 (58314)	0.5 (4318)	0.7 (144)
Pre-gestational diabetes						
No	99.6 (2 688 552)	99.6 (2 665 957)	99.2 (22 595)	95.1 (844 483)	93.7 (810 695)	84.5 (18 601)
Yes	0.4 (11 123)	0.4 (10 943)	0.8 (180)	4.9 (43 099)	6.3 (54 877)	15.5 (3409)

CVD, cardiovascular disease.

a Mother’s region of birth and marital status were categorized differently in British Columbia, Canada.

b Information on maternal education and smoking was not available in British Columbia, Canada.

During the observation period from 1991 to 2020 in Sweden (median age at the end of follow-up: 13–15 years), 154 399 children were diagnosed with ADHD (rate 4.1/1000 child-years), 64 436 with ASD (rate 1.7/1000 child-years) and 22 758 with ID (rate 0.6/1000 child-years). In BC, the corresponding numbers of children with ADHD, ASD and ID during 1992–2020 (median age at the end of follow-up: 12–13 years) were 78 333 (rate 7.1/1000 child-years), 19 018 (rate 1.6/1000 child-years) and 4132 (rate 0.4/1000 child-years), respectively. The probability of staying free from ADHD and ASD during follow-up decreased faster among the offspring exposed to maternal CVD compared with offspring without maternal CVD (Supplementary Figure S3, available as Supplementary data at *IJE* online). The Cox models showed that maternal CVD was associated with increased hazards of ADHD and ASD in both Sweden and BC, although the magnitude of the associations was attenuated to some extent after adjustment for maternal characteristics (Table 2).

**Table 2.** Incidence rates and hazard ratios of neurodevelopmental disorders according to pre-existing maternal and paternal cardiovascular diseases: singleton offspring live-born without major malformations in Sweden 1990 to 2019 and in British Columbia, Canada, 1992 to 2019

	ADHD				ASD				ID			
	No. of events	**Rate** ^a^	**Model 1 HR (95% CI)** ^b^	**Model 2 HR (95% CI)** ^b^	No. of events	**Rate** ^a^	**Model 1 HR (95% CI)** ^b^	**Model 2 HR (95% CI)** ^b^	No. of events	**Rate** ^a^	**Model 1 HR (95% CI)** ^b^	**Model 2 HR (95% CI)** ^b^
**Sweden**												
**Maternal CVD (*n*=2 699 675)**												
Composite maternal CVD												
No	153 355	4.1	1.00 (Ref.)	1.00 (Ref.)	63 974	1.7	1.00 (Ref.)	1.00 (Ref.)	22 626	0.6	1.00 (Ref.)	1.00 (Ref.)
Yes	1044	5.4	1.29 (1.21–1.37)	1.17 (1.10–1.25)	462	2.3	1.21 (1.10–1.32)	1.12 (1.02–1.23)	132	0.7	1.06 (0.90–1.26)	0.99 (0.83–1.18)
Subtype of maternal CVD^c^												
Cerebrovascular disease	204	6.0	1.40 (1.22–1.61)	1.21 (1.06–1.39)	99	2.9	1.54 (1.26–1.88)	1.35 (1.11–1.65)	28	0.8	1.30 (0.90–1.88)	1.12 (0.77–1.62)
Arrhythmia	565	5.2	1.24 (1.14–1.34)	1.16 (1.07–1.26)	242	2.2	1.11 (0.97–1.26)	1.04 (0.91–1.18)	57	0.5	0.81 (0.63–1.05)	0.79 (0.61–1.03)
Heart failure	26	6.4	2.04 (1.08–2.34)	1.44 (0.98–2.12)	18	4.4	2.12 (1.30–3.46)	1.92 (1.18–3.14)	<5^d^	0.9	1.54 (0.58–4.11)	1.17 (0.44–3.10)
Valvular heart disease	85	4.9	1.15 (0.93–1.43)	1.12 (0.91–1.39)	41	2.3	1.20 (0.88–1.65)	1.15 (0.84–1.57)	19	1.1	1.73 (1.10–2.71)	1.49 (0.95–2.33)
Congenital heart disease	239	5.4	1.35 (1.19–1.54)	1.17 (1.03–1.33)	108	2.4	1.24 (1.02–1.51)	1.15 (0.94–1.39)	35	0.8	1.26 (0.90–1.75)	1.15 (0.83–1.61)
**Paternal CVD (*n*=2 672 229)**												
Composite paternal CVD												
No	151 794	4.1	1.00 (Ref.)	1.00 (Ref.)	63 281	1.7	1.00 (Ref.)	1.00 (Ref.)	22 367	0.6	1.00 (Ref.)	1.00 (Ref.)
Yes	1212	4.7	1.10 (1.04–1.16)	1.07 (1.01–1.13)	572	2.2	1.13 (1.04–1.22)	1.08 (1.00–1.17)	185	0.7	1.13 (0.98–1.30)	1.08 (0.94–1.25)
Subtype of paternal CVD^c^												
Cerebrovascular disease	254	5.8	1.36 (1.20–1.54)	1.29 (1.4–1.46)	103	2.3	1.22 (1.00–1.48)	1.13 (0.93–1.37)	34	0.8	1.24 (0.88–1.74)	1.09 (0.78–1.53)
Arrhythmia	710	4.3	1.02 (0.94–1.09)	1.00 (0.93–1.08)	349	2.1	1.09 (0.98–1.21)	1.06 (0.95–1.17)	100	0.6	0.96 (0.80–1.18)	0.96 (0.79–1.17)
Heart failure	55	5.2	1.23 (0.94–1.60)	1.12 (0.86–1.46)	24	2.3	1.14 (0.77–1.70)	0.99 (0.66–1.48)	11	1.0	1.62 (0.90–2.93)	1.24 (0.64–2.23)
Valvular heart disease	121	4.2	0.98 (0.82–1.17)	0.99 (0.83–1.19)	58	2.0	1.03 (0.80–1.34)	1.00 (0.77–1.30)	24	0.8	1.33 (0.89–1.98)	1.20 (0.81–1.80)
Congenital heart disease	185	4.9	1.20 (1.04–1.39)	1.10 (0.95–1.27)	96	2.5	1.30 (1.06–1.58)	1.24 (1.01–1.51)	29	0.7	1.21 (0.84–1.74)	1.17 (0.81–1.68)
**British Columbia, Canada**												
**Maternal CVD (*n*=887 582)**												
Composite maternal CVD												
No	76 290	7.0	1.00 (Ref.)	1.00 (Ref.)	18 404	1.6	1.00 (Ref.)	1.00 (Ref.)	4035	0.3	1.00 (Ref.)	1.00 (Ref.)
Yes	2043	9.5	1.29 (1.23–1.35)	1.14 (1.09–1.20)	614	2.7	1.28 (1.17–1.39)	1.17 (1.07–1.27)	97	0.4	1.18 (0.96–1.44)	1.08 (0.89–1.32)
Subtype of maternal CVD^c^												
Cerebrovascular disease	195	10.1	1.41 (1.21–1.64)	1.19 (1.02–1.39)	57	2.8	1.29 (0.95–1.74)	1.10 (0.81–1.48)	13	0.6	1.79 (1.04–3.09)	1.57 (0.91–2.70)
Arrhythmia	1274	9.4	1.25 (1.18–1.33)	1.12 (1.06–1.19)	407	2.9	1.29 (1.16–1.43)	1.18 (1.06–1.31)	63	0.4	1.21 (0.95–1.56)	1.13 (0.88–1.45)
Heart failure	68	10.9	1.47 (1.13–1.91)	1.27 (0.97–1.65)	19	2.9	1.22 (0.75–1.98)	1.07 (0.66–1.74)	<5^d^	0.6	1.65 (0.62–4.40)	1.38 (0.52–3.68)
Valvular heart disease	300	8.2	1.16 (1.03–1.31)	1.12 (0.99–1.26)	73	1.9	1.22 (0.95–1.56)	1.14 (0.89–1.46)	9	0.2	0.65 (0.34–1.25)	0.65 (0.34–1.24)
Congenital heart disease	390	10.5	1.44 (1.29–1.61)	1.18 (1.06–1.32)	117	3.0	1.30 (1.07–1.59)	1.19 (0.98–1.45)	18	0.5	1.28 (0.80–2.02)	1.07 (0.68–1.70)
**Paternal CVD** (***n*=811 481)**												
Composite paternal CVD												
No	71 270	7.0	1.00 (Ref.)	1.00 (Ref.)	16 911	1.6	1.00 (Ref.)	1.00 (Ref.)	3778	0.4	1.00 (Ref.)	1.00 (Ref.)
Yes	1066	8.0	1.07 (1.01–1.14)	0.99 (0.93–1.06)	322	2.3	1.06 (0.94–1.19)	1.00 (0.89–1.12)	52	0.4	1.03 (0.78–1.36)	0.99 (0.75–1.31)
Subtype of paternal CVD^c^												
Cerebrovascular disease	150	9.5	1.29 (1.09–1.52)	1.14 (0.96–1.35)	57	3.4	1.58 (1.21–2.06)	1.42 (1.09–1.86)	9	0.5	1.51 (0.78–2.90)	1.34 (0.70–2.57)
Arrhythmia	677	7.4	0.99 (0.91–1.07)	0.93 (0.86–1.01)	219	2.3	1.05 (0.92–1.21)	1.00 (0.87–1.16)	27	0.3	0.78 (0.53–1.14)	0.77 (0.53–1.12)
Heart failure	61	9.3	1.31 (1.00–1.73)	1.21 (0.92–1.58)	16	2.3	1.11 (0.68–1.81)	1.01 (0.62–1.64)	<5^d^	0.3	0.83 (0.21–3.30)	0.71 (0.18–2.84)
Valvular heart disease	125	8.4	1.10 (0.91–1.33)	1.02 (0.85–1.24)	33	2.1	1.05 (0.73–1.50)	0.98 (0.69–1.41)	7	0.4	1.21 (0.58–2.53)	1.15 (0.55–2.42)
Congenital heart disease	196	9.0	1.23 (1.06–1.42)	1.02 (0.88–1.19)	47	2.0	0.84 (0.62–1.14)	0.80 (0.59–1.08)	10	0.4	1.24 (0.63–2.43)	1.10 (0.56–2.15)

ADHD, attention-deficit/hyperactivity disorder; ASD, autism spectrum disorder; CI, confidence interval; CVD, cardiovascular disease; HR, hazard ratio; ID, intellectual disability.

a Incidence rate per 1000 child-years.

b Model 1 was minimally adjusted for birth year and sex. Model 2 was further adjusted for mother’s age at delivery, parity, education (Sweden only), region of birth, marital status/cohabitation with partner, smoking during early pregnancy (Sweden only), pre-gestational diabetes, pre-gestational hypertension and parental history of any neurodevelopmental or psychiatric disorders. The estimates for paternal CVD in Model 2 were additionally adjusted for maternal CVD.

c All CVD subtypes were coded as binary: no (reference)/yes.

d Exact count is suppressed for confidentiality reasons.

**Figure 1.**
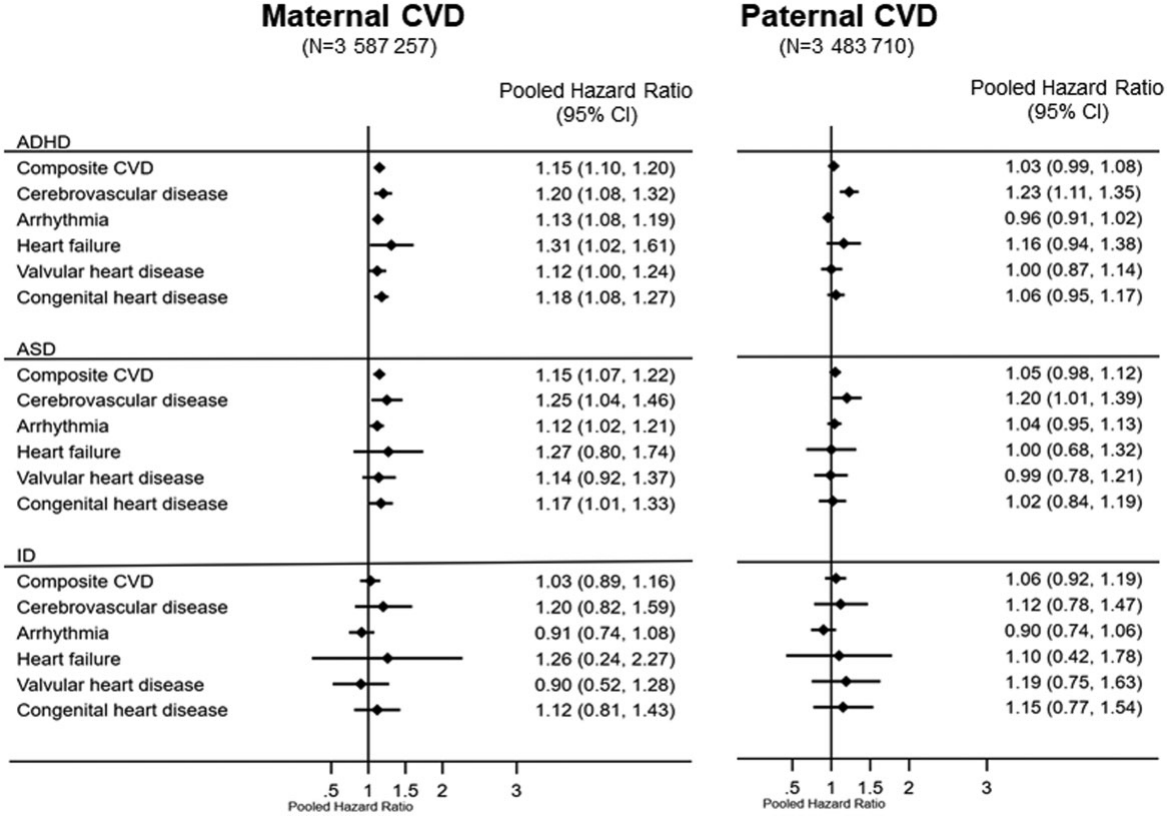
Plots showing pooled adjusted hazard ratios of neurodevelopmental disorders according to pre-existing maternal and paternal cardiovascular diseases: singleton offspring live-born without major malformations in Sweden 1990 to 2019 and in British Columbia, Canada, 1992 to 2019. ADHD, attention-deficit/hyperactivity disorder; ASD, autism spectrum disorder; CI, confidence interval; CVD, cardiovascular disease; ID, intellectual disability; NDD, neurodevelopmental disorder. The pooled hazard ratios represent the estimates combined by meta-analysing the pre-calculated hazard ratios and standard errors in the two countries. The hazard ratios were adjusted for child’s sex and birth year, and mother’s age at delivery, parity, education (Sweden only), region of birth, marital status/cohabitation with partner, smoking during early pregnancy (Sweden only), pre-gestational diabetes, pre-gestational hypertension and parental history of any neurodevelopmental or psychiatric disorders. The hazard ratios for paternal CVD were further adjusted for maternal CVD

The meta-analysis suggested that maternal CVD, overall, was associated with 15% higher hazard of both ADHD (HR 1.15, 95% CI 1.10–1.20, I^2^ 0%, *P* for heterogeneity 0.54; Figure 1) and ASD (HR 1.15, 95% CI 1.02–1.23, I^2^ 0%, *P* for heterogeneity 0.50) in offspring, when compared with no maternal CVD. However, no association was found between maternal CVD and ID in offspring (HR 1.03, 95% CI 0.89–1.16, I^2^ 0%, *P* for heterogeneity 0.53).

Among the subtypes of maternal CVD, increased hazard of ADHD in offspring was found for all included subtypes, namely maternal heart failure (HR 1.31, 95% CI 1.02–1.61), cerebrovascular disease (HR 1.20, 95% CI 1.08–1.32), congenital heart disease (HR 1.18, 95% CI 1.08–1.27), arrhythmia (HR 1.13, 95% CI 1.08–1.19) and valvular heart disease (HR 1.12, 95% CI 1.00–1.24), when compared with offspring of mothers without the respective CVD condition. Offspring exposed to maternal cerebrovascular disease, congenital heart disease and arrythmia had elevated HRs of ASD, compared with non-exposed offspring. None of the maternal CVD subtypes was associated with ID in offspring (Table 2).

Paternal CVD showed associations, albeit much weaker compared with maternal CVD, with ADHD and ASD in Sweden, but not in BC (Table 2). The meta-analysis, however, suggested no associations between paternal CVD and HRs of ADHD, ASD or ID in offspring (Figure 1). When the analysis was stratified by subtypes of paternal CVD, offspring exposed to paternal cerebrovascular disease showed increased hazard of ADHD (HR 1.23, 95% CI 1.11–1.35) and ASD (HR 1.20, 95% CI 1.01–1.39).

In the stratified analysis by offspring’s sex, the incidence rates of NDD outcomes were higher in males than in females in both countries (Table 3). In the Swedish cohort, maternal CVD was strongly associated with ADHD and ASD in males, but not in females. In BC, however, maternal CVD showed clear associations with ADHD and ASD in both sexes, with a stronger association with ASD in females compared with males.

**Table 3.** Sex-stratified incidence rates and hazard ratios of neurodevelopmental disorders according to pre-existing maternal and paternal cardiovascular disease: singleton offspring live-born without major malformations in Sweden 1990 to 2019 and in British Columbia, Canada, 1992 to 2019

	ADHD				ASD				ID			
	No. of events	**Rate** ^a^	**Model 1 HR (95% CI)** ^b^	**Model 2 HR (95% CI)** ^b^	No. of events	**Rate** ^a^	**Model 1 HR (95% CI)** ^b^	**Model 2 HR (95% CI)** ^b^	No. of events	**Rate** ^a^	**Model 1 HR (95% CI)** ^b^	**Model 2 HR (95% CI)** ^b^
**Sweden**												
**Male (*n*=1 380 710)**												
Composite maternal CVD												
No	95 035	4.9	1.00 (Ref.)	1.00 (Ref.)	42 413	2.2	1.00 (Ref.)	1.00 (Ref.)	13 987	0.7	1.00 (Ref.)	1.00 (Ref.)
Yes	705	7.1	1.32 (1.22–1.42)	1.21 (1.12–1.30)	326	3.2	1.21 (1.08–1.35)	1.14 (1.02–1.27)	80	0.8	1.00 (0.80–1.24)	0.93 (0.75–1.16)
Composite paternal CVD												
No	94 015	4.9	1.00 (Ref.)	1.00 (Ref.)	41 638	2.2	1.00 (Ref.)	1.00 (Ref.)	13 718	0.7	1.00 (Ref.)	1.00 (Ref.)
Yes	820	6.2	1.13 (1.05–1.21)	1.10 (1.03–1.18)	403	3.0	1.14 (1.03–1.26)	1.09 (0.99–1.21)	123	0.9	1.16 (0.97–1.39)	1.12 (0.94–1.34)
**Female (*n*=1 318 891)**												
Composite maternal CVD												
No	58 320	3.1	1.00 (Ref.)	1.00 (Ref.)	21 561	1.1	1.00 (Ref.)	1.00 (Ref.)	8639	0.5	1.00 (Ref.)	1.00 (Ref.)
Yes	339	3.5	1.23 (1.11–1.37)	1.10 (0.99–1.23)	136	1.4	1.19 (1.00–1.40)	1.08 (0.91–1.28)	52	0.5	1.19 (0.90–1.56)	1.09 (0.83–1.44)
Composite paternal CVD												
No	57 779	3.1	1.00 (Ref.)	1.00 (Ref.)	21 206	1.1	1.00 (Ref.)	1.00 (Ref.)	8490	0.5	1.00 (Ref.)	1.00 (Ref.)
Yes	392	3.1	1.04 (0.94–1.15)	1.00 (0.91–1.11)	173	1.3	1.12 (0.97–1.31)	1.07 (0.92–1.25)	63	0.5	1.08 (0.84–1.39)	1.02 (0.80–1.31)
*P* for interaction between maternal CVD and offspring sex			0.001	0.001			0.048	0.045			0.783	0.761
*P* for interaction between paternal CVD and offspring sex			0.001	0.001			0.060	0.065			0.236	0.240
**British Columbia, Canada**												
**Male (*n*=451 770)**												
Composite maternal CVD												
No	53 231	9.9	1.00 (Ref.)	1.00 (Ref.)	14 223	2.5	1.00 (Ref.)	1.00 (Ref.)	2606	0.4	1.00 (Ref.)	1.00 (Ref.)
Yes	1434	13.3	1.28 (1.21–1.35)	1.14 (1.08–1.21)	449	3.9	1.20 (1.09–1.33)	1.12 (1.01–1.23)	57	0.5	1.05 (0.81–1.36)	0.97 (0.75–1.26)
Composite paternal CVD												
No	49 667	9.8	1.00 (Ref.)	1.00 (Ref.)	13 053	2.4	1.00 (Ref.)	1.00 (Ref.)	2418	0.4	1.00 (Ref.)	1.00 (Ref.)
Yes	749	11.3	1.07 (0.99–1.15)	0.99 (0.92–1.07)	245	3.5	1.04 (0.92–1.19)	1.00 (0.88–1.14)	43	0.6	1.31 (0.96–1.78)	1.26 (0.92–1.71)
**Female (*n*=435 812)**												
Composite maternal CVD												
No	23 048	4.2	1.00 (Ref.)	1.00 (Ref.)	4181	0.7	1.00 (Ref.)	1.00 (Ref.)	1429	0.2	1.00 (Ref.)	1.00 (Ref.)
Yes	610	5.6	1.31 (1.21–1.42)	1.14 (1.05–1.24)	165	1.5	1.54 (1.31–1.81)	1.34 (1.14–1.57)	40	0.3	1.43 (1.05–1.96)	1.30 (0.95–1.78)
Composite paternal CVD												
No	21 594	4.2	1.00 (Ref.)	1.00 (Ref.)	3858	0.7	1.00 (Ref.)	1.00 (Ref.)	1360	0.2	1.00 (Ref.)	1.00 (Ref.)
Yes	316	4.7	1.08 (0.97–1.21)	0.99 (0.88–1.11)	77	1.1	1.10 (0.87–1.39)	1.00 (0.79–1.27)	9	0.1	0.51 (0.27–0.99)	0.50 (0.26–0.96)
*P* for interaction between maternal CVD and offspring sex			0.706	0.772			0.010	0.010			0.215	0.187
*P* for interaction between paternal CVD and offspring sex			0.558	0.581			0.748	0.776			0.007	0.007

ADHD, attention-deficit/hyperactivity disorder; ASD, autism spectrum disorder; CI, confidence interval; CVD, cardiovascular disease; HR, hazard ratio; ID, intellectual disability.

a Incidence rate per 1000 child-years.

b Model 1 was minimally adjusted for birth year. Model 2 was further adjusted for mother’s age at delivery, parity, education (Sweden only), region of birth, marital status/cohabitation with partner, smoking during early pregnancy (Sweden only), pre-gestational diabetes, pre-gestational hypertension and parental history of any neurodevelopmental or psychiatric disorders. The estimates for paternal CVD in Model 2 were additionally adjusted for maternal CVD.

The natural indirect effects in the causal mediation analysis (Table 4) indicated that the total effect of maternal CVD on ADHD or ASD in offspring was not considerably mediated through preterm birth.

**Table 4.** Sex-stratified causal mediation analysis to estimate the impact of preterm delivery on the association between maternal pre-existing cardiovascular disease and offspring’s ADHD and ASD: singleton offspring live-born without major malformations in Sweden 1990 to 2019 and British Columbia, Canada, 1992 to 2019

	ADHD	ASD
	IRR (95% CI)	IRR (95% CI)
**Sweden**		
**Male (*n*=1 380 710)**		
Total effect (TE)	1.20 (1.11–1.29)	1.13 (1.01–1.26)
Natural direct effect (NDE)^a^	1.19 (1.10–1.28)	1.12 (1.00–1.25)
Natural indirect effect (NIE)	1.01 (1.00–1.01)	1.01 (1.00–1.02)
Controlled direct effect (CDE)^b^	1.46 (1.17–1.83)	1.39 (1.00–1.92)
Mediated proportion^c^	5%	7%
**Female (*n*=1 318 891)**		
Total effect (TE)	1.09 (0.98–1.22)	1.06 (0.89–1.25)
Natural direct effect (NDE)^a^	1.09 (0.98–1.22)	1.04 (0.87–1.23)
Natural indirect effect (NIE)	1.00 (0.99–1.00)	1.02 (1.00–1.03)
Controlled direct effect (CDE)^b^	1.03 (0.70–1.50)	1.71 (1.10–1.67)
Mediated proportion^c^	0%	34%
**British Columbia, Canada**		
**Male (*n*=451 770)**		
Total effect (TE)	1.13 (1.08–1.20)	1.12 (1.01–1.22)
Natural direct effect (NDE)^a^	1.13 (1.07–1.19)	1.12 (1.01–1.23)
Natural indirect effect (NIE)	1.00 (1.00–1.00)	1.00 (0.99–1.00)
Controlled direct effect (CDE)^b^	1.13 (1.07–1.20)	1.14 (1.03–1.25)
Mediated proportion^c^	0%	0%
**Female (*n*=435 812)**		
Total effect (TE)	1.14 (1.05–1.23)	1.32 (1.12–1.53)
Natural direct effect (NDE)^a^	1.14 (1.05–1.23)	1.32 (1.11–1.53)
Natural indirect effect (NIE)	1.00 (0.99–1.00)	1.00 (1.00–1.01)
Controlled direct effect (CDE)^b^	1.14 (1.04–1.23)	1.33 (1.11–1.54)
Mediated proportion^c^	0%	0%

ADHD, attention-deficit/hyperactivity disorder; ASD, autism spectrum disorder; CI, confidence interval; CVD, cardiovascular disease; IRR, incidence rate ratio.

All parameters were derived from Poisson regression models and were conditional on the following covariates: child’s birth year; mother’s age at delivery, parity, education (Sweden only), region of birth, marital status/cohabitation with partner, smoking during early pregnancy (Sweden only), pre-gestational diabetes, pre-gestational hypertension and parental history of any neurodevelopmental or psychiatric disorders.

a The NDE represents the effect of maternal CVD in the absence of preterm birth.

b The CDE represents the effect of maternal CVD obtained by setting the value of preterm birth to 1 (i.e. everyone is assumed to be preterm).

c The proportion mediated was calculated using the formula: [IRR^NDE^ (IRR^NIE^− 1)/(IRR^NDE^ * IRR^NIE^− 1)]*100.

In the sensitivity analyses, omitting maternal education and smoking from the fully adjusted models (Supplementary Table S4, available as Supplementary data at *IJE* online) or additional adjustment for maternal BMI in Sweden (Supplementary Table S5, available as Supplementary data at *IJE* online) or parental neighbourhood income quintiles in BC (Supplementary Table S6, available as Supplementary data at *IJE* online) did not influence the results considerably. Multiple imputation of missing data (Supplementary Table S7, available as Supplementary data at *IJE* online) and analyses restricted to later-born cohorts (Supplementary Tables S8 and S9, available as Supplementary data at *IJE* online) produced results consistent with the main analyses. The associations between maternal CVD and offspring ASD remained robust when excluding ID diagnoses (Supplementary Table S10, available as Supplementary data at *IJE* online). Paternal comparison models showed largely unchanged associations when adjusting for paternal age at childbirth (Supplementary Figure S4, available as Supplementary data at *IJE* online). Including paternal CVD as an additional adjustment did not change the magnitude of the association of maternal CVD and ADHD and ASD (data not shown).

## Discussion

In this cohort study including nearly 3.6 million children born in Sweden or BC, Canada, we found that pre-pregnancy maternal CVD was associated with increased risks of ADHD and ASD in offspring. The causal mediation analyses suggested that the associations were largely independent of preterm delivery. We found no excess risk of ID among offspring exposed to maternal CVD. Paternal CVD was not generally associated with offspring risks of NDD outcomes.

To our knowledge, this is the first population-based study to report the risk of NDDs in children of mothers with pre-existing cardiovascular conditions. Our results are consistent with the existing literature documenting elevated risks of NDDs in children exposed to maternal cardiovascular risk factors including obesity,^10^ hypertension^15^^,^^16^ and diabetes.^14^ A meta-analysis reported higher risks of ASD and ADHD among children exposed to maternal hypertensive disorders, although most of the included studies did not adequately control for confounding.^16^

The association between maternal CVD and offspring risk of ADHD and ASD in our study persisted even after adjustment for parental history of any NDD or psychiatric disorders as well as a number of other covariates related to maternal social and health characteristics. The genetic factors or shared familiar factors are a less likely explanation, as there was no association with paternal CVD. Among paternal CVD subtypes, only cerebrovascular disease showed an increased risk of ADHD and ASD in offspring. We hypothesize that this could be due to the heightened stress experienced by the caregiving mother (i.e. spillover effect), particularly in cases of severe cerebrovascular disease like stroke.^33^^,^^34^ Our results thus suggest that the maternal intrauterine environment plays a more significant role than genetics in the underlying mechanism linking CVD and NDDs. Furthermore, the associations between maternal CVD and offspring ADHD and ASD were more pronounced in males compared with females. This is in line with animal experiments suggesting that maladaptive placental responses to maternal-fetal stressors are sex dependent, with male fetuses being more susceptible to the development of NDD.^35^^,^^36^

We observed minimal mediating effect of preterm birth, consistent with previous research showing that the increased risks of ASD and ADHD in children of mothers born with hypertensive disorders during pregnancy were independent of fetal growth and gestational age at birth.^15^ However, studying gestational age/preterm birth as a mediator is inherently challenging since it might be caused by various known and unknown maternal pathological processes (e.g. placental abruption, preeclampsia, gestational diabetes and obesity). If not properly accounted for, these factors may violate the mediator-outcome confounding assumption and induce collider bias.^37^^,^^38^ Further research is needed to conduct more detailed analyses of the casual mechanisms and pathways.

Preconceptional CVD in women may lead to various physiological disturbances during pregnancy, including abnormal uteroplacental perfusion, hormonal imbalances, metabolic dysfunction, increased inflammation and oxidative stress.^39^^,^^40^ These disturbances may negatively impact on the structural brain development in the offspring. Existing evidence suggests a link between pre-existing cardiac dysfunction and compromised placental circulation, which in turn contributes to pregnancy-related and neonatal complications.^41^^,^^42^ Additionally, the use of CVD medication during pregnancy is another possible mechanism, as fetal brain development is particularly sensitive to medication exposure.^43^

### Strengths and limitations

The strengths of our study include the use of data from several population-based registers in two countries, with almost 30 years of follow-up. This allowed for robust estimation and cross-validation of the associations between various maternal CVD conditions and NDD outcomes in offspring. We accounted for several important maternal confounders, including smoking, NDD and psychiatric conditions, and also employed a family-based study design to investigate potential unmeasured genetic confounding. The Swedish population registers are generally known to have high quality and nationwide coverage,^22^^,^^23^ minimizing selection bias due to systematic losses to follow-up. Additionally, the ethnically diverse and heterogeneous nature of the BC cohort^44^ enhanced the generalizability of our findings.

Some limitations of the study should be acknowledged. First, despite adjusting for parental history of NDD and psychiatric comorbidities, residual confounding may persist due to possible underdiagnoses in the parents. Second, although the study had adequate statistical power to estimate the associations of interest, there were insufficient cases in some CVD subgroups, which might obscure true associations, particularly with ID. Third, data on CVD and NDD in Sweden came from specialist settings, with outpatient diagnoses available only from 2001. This might have resulted in milder cases being under-represented. Whereas our BC data came from both specialized and primary care settings, concerns exist regarding the reliability of ASD diagnoses based on BC health administrative data which may not consistently distinguish between ASD and other NDDs,^45^ unlike the reported 94% accuracy in Sweden.^46^ Increased awareness and improvements in diagnostic criteria over time could also lead to exposure and outcomes being misclassified, particularly in the early years of follow-up. Such potential misclassifications are expected to be non-differential.

## Conclusion

This large population-based cohort study revealed that pre-existing maternal CVD might be a risk factor for ADHD and ASD in offspring. The observed risks appear to be largely unexplained by paternal CVD, suggesting a potential involvement of intrauterine mechanisms. Although the precise mechanisms need to be carefully assessed in future studies, the findings highlight the importance of close clinical monitoring and support to reproductive-aged women with an underlying CVD, for prevention of or early intervention for ADHD and ASD in their children.

## Ethics approval

The study was approved by the Ethics Review Authority in Sweden (approval number 2020–01545) and the Ethics Committee at the University of British Columbia, Canada (approval number H20-00902).

## Supplementary Material

dyad184_Supplementary_Data

## Data Availability

De-identified Swedish data can be requested via application with a methodologically sound research proposal. Such a request can be sent to Dr Neda Raza [neda.razaz@ki.se] for consideration by the ethics review committee. Access to data from British Columbia, Canada, used in this study is provided by the Data Steward(s) and is subject to approval. These data can be requested for research projects through the Data Steward(s) or their designated service providers. All inferences, opinions and conclusions drawn in this publication are those of the authors alone.
