# Supplementary material for: Association of pre-existing maternal cardiovascular diseases with neurodevelopmental disorders in offspring: a cohort study in Sweden and British Columbia, Canada
Source: Int J Epidemiol. 2023 Dec 27;53(1):dyad184. doi: 10.1093/ije/dyad184 (PMC10859157; doi:10.1093/ije/dyad184)
Supplement: dyad184_Supplementary_Data [file dyad184_supplementary_data.pdf]

Supplementary Online Content

Association of pre-existing maternal cardiovascular diseases with neurodevelopmental disorders in offspring: a cohort study in Sweden and British Columbia, Canada

Supplementary Methods S1: Description of registers

Supplementary Methods S2: Causal mediation analysis

Supplementary Methods S3: Multiple imputation of missing data

Supplementary Figure S1. Flow diagram of the study samples.

Supplementary Figure S2. A conceptual framework of the study.

Supplementary Figure S3. Kaplan-Meier survival curves showing event-free survival probabilities by maternal cardiovascular disease status: singleton offspring live-born without major malformations in Sweden 1990 to 2019 (N=2 699 675).

Supplementary Figure S4. Hazard ratios of neurodevelopmental disorders according to paternal cardiovascular disease, adjusted for paternal age at child's birth: singleton offspring live-born without major malformations in Sweden 1990 to 2019 (N=2 672 229) and British Columbia, Canada 1992 to 2019 (N=741 026).

Supplementary Table S1. Register databases from which the study variables were derived.

Supplementary Table S2. International Classification of Disease (ICD) codes used to define maternal diseases and offspring neurodevelopmental disorders.

Supplementary Table S3. Sex-stratified incidence rates and hazard ratios of neurodevelopmental disorders according to preterm birth: singleton offspring live-born without major malformations in Sweden 1990 to 2019 and in British Columbia, Canada 1992 to 2019.

Supplementary Table S4. Incidence rates and hazard ratios of neurodevelopmental disorders according to pre-existing maternal cardiovascular diseases, with versus without adjustment for maternal education and smoking: singleton offspring live-born without major malformations in Sweden 1990 to 2019 (N=2 699 675).

Supplementary Table S5. Incidence rates and hazard ratios of neurodevelopmental disorders according to pre-existing maternal cardiovascular diseases, adjusted for maternal early pregnancy body mass index: singleton offspring live-born without major malformations in Sweden 1992 to 2019 (N=2 311 311).

Supplementary Table S6. Incidence rates and hazard ratios of neurodevelopmental disorders according to pre-existing maternal cardiovascular diseases, adjusted for parental neighbourhood income quintiles: singleton offspring live-born without major malformations in British Columbia, Canada 1992 to 2019 (N= 863 440).

Supplementary Table S7. Multiple-imputation analysis of the associations between maternal pre-existing cardiovascular diseases and offspring's neurodevelopmental disorders: singleton offspring live-born without major malformations in Sweden 1990 to 2019 (N=2 940 626).

Supplementary Table S8. Sensitivity analysis restricted to later-born cohorts, concerning the associations between maternal pre-existing cardiovascular diseases and offspring's neurodevelopmental disorders: singleton offspring live-born without major malformations in Sweden 1997 to 2019 (N=1 960 441).

Supplementary Table S9. Sensitivity analysis restricted to later-born cohorts, concerning the associations between pre-existing maternal cardiovascular diseases and offspring's neurodevelopmental disorders: singleton offspring live-born without major malformations in British Columbia, Canada 2001 to 2019 (N= 589 933).

Supplementary Table S10. Incidence rates and hazard ratios of autism spectrum disorder without intellectual disability according to pre-existing maternal cardiovascular diseases: singleton offspring live-born without major malformations in Sweden 1990 to 2019 (N=2 672 229) and British Columbia, Canada 1992 to 2019 (N= 887 582).

Supplementary Methods S1: Description of registers

A detailed description of all register databases used in the study is given below:

<u>The Swedish Medical Birth Register</u>

The Swedish Medical Birth Register (MBR) retrieves data on prenatal, obstetric, and neonatal characteristics, including maternal diagnoses, based on medical records from the prenatal care, the delivery care, and the neonatal care. Founded in 1973, the MBR covers >98% of all births occurring in Sweden[1].

<u>The Swedish National Patient Register</u>

The National Patient Register (NPR) consists of the inpatient and outpatient registers and holds data on health care from both public and private caregivers. The inpatient register, also known as hospital discharge register, was initiated in 1964 (psychiatric diagnoses since 1973), but complete nationwide coverage was obtained in 1987. The outpatient register was launched in 2001 and records information on visits to specialized outpatient care. The diagnoses of diseases recorded in the NPR are identified using the Swedish version of the International Classification of Disease (ICD) codes[2].

<u>The Swedish Cause of Death Register</u>

The Swedish Cause of Death Register (CDR) is an excellent source of data for register-based research and contains information on all deaths in Sweden since 1952. The register is known for its high quality and complete data and is linked to other national registers through unique personal identification numbers[3]. Unlike the NPR that uses the Swedish version of ICD codes to classify diseases, the CDR uses the international version of the classification system to facilitate international comparison of cause-specific mortality statistics[4].

<u>The Swedish Total Population Register</u>

Since 1968, the Total Population Register (TPR) contains sociodemographic information (eg, sex, country of birth) as well as data on important life events including dates of birth, death and migration of all residents in Sweden. The TPR allows complete follow up and censoring of individuals and thereby minimizes the risk of selection bias[5].

<u>The Swedish Education Register</u>

Since 1985, the Education Register in Sweden contains annually updated data on completed education, collected from more than 30 different sources including the schools and education providers and questionnaire surveys. Data on education is coded according to the Swedish

education nomenclature SUN, a classification system which was later adapted to the International Standard Classification of Education (ISCED 97) in 2000, distinguishing between the level and type of education[6]

The Swedish Prescribed Drug Register

The Prescribed Drug Register (PDR) was established in July 2005 and contains data on all outpatient prescribed drugs dispensed at pharmacies throughout Sweden. The data on the drug prescriptions recorded in the PDR is retrieved from the Swedish eHealth Agency. The overall quality of the data in the register is good, with minimal errors and incompletion. The World Health Organization's Anatomical Therapeutic Chemical (ATC) classification system is used to classify all drugs in the PDR[7].

The BC Vital Statistics

The BC Vital Statistics database contains both birth and death files. The BC Vital Statistics Birth database covers >98% of all births in British Columbia (BC) and contains information on maternal characteristics, diseases, pregnancy, delivery and neonatal complications, and birth characteristics from January 1, 1985[8]. On the other hand, information on all deaths occurring in BC since January 1, 1985 is retrieved from the BC Vital Statistics Death file[9].

The BC Discharge Abstract Database

The Discharge Abstract Database (DAD) captures administrative, demographic and clinical data of in-patients in BC since April 1, 1985, including information of hospital admission and discharge dates, day surgery, and diagnoses using ICD-9/10 codes[10].

The BC Medical Services Plan

Established in 1965, the BC Medical Services Plan (MSP) a provincial health insurance program covering all eligible residents in BC. The MSP files contain both specialist and primary care physician billings data covering all fee-for-service outpatient claims, including service date and associated diagnoses based on ICD-9 codes from April 1, 1991 onwards[11].

The BC PharmaNet

PharmaNet is province-wide electronic medical record system containing information on all outpatient prescriptions dispensed from community pharmacies in BC from January 1, 1996[12]. Information includes the dispensation date, drug name or unique Drug Identification Numbers, which are mapped to ATC classification system.

The BC Central Demographics File

The Central Demographics File, previously known as the Consolidation File, provides basic demographic (eg, age and sex) and registration data for provincial health coverage from January 1, 1986 onward.

Supplementary Methods S2: Causal mediation analysis

The identification of the natural direct and indirect effects in the causal mediation analysis relies on the assumptions that there were no unmeasured confounders affecting (i) the exposure-outcome association, (ii) the exposure-mediator association, (iii) the mediator-outcome association, and (iv) no unmeasured mediator-outcome confounder affected by the exposure[13]. We treated child's birth year as a mediator-outcome confounder while the baseline maternal characteristics were used as exposure-outcome and exposure-mediator confounders.

Supplementary Methods S3: Multiple imputation of missing data
Children with missing data on any study variables were excluded (Sweden 8.2%; BC 6.2%) from the main analysis. In a supplementary analysis of the Swedish data, we created 10 imputed datasets under the assumption of missing at random, using the multiple imputation by chained equations procedure[14]. The estimates from the imputed datasets were combined by Rubin's rule.

Supplementary References

1. Swedish National Board of Health and Welfare. *Det Statistiska Registrets Framställning Och Kvalitet: Medicinska Födelseregistret (Production and Quality of the statistical Register: Medical Birth Register*.; 2021. Accessed December 28, 2021. https://www.socialstyrelsen.se/globalassets/sharepoint-dokument/artikelkatalog/statistik/2021-9-7547.pdf

2. Ludvigsson JF, Andersson E, Ekbom A, et al. External review and validation of the Swedish national inpatient register. *BMC Public Health*. 2011;11. doi:10.1186/1471-2458-11-450

3. Ludvigsson JF, Otterblad-Olausson P, Pettersson BU, Ekbom A. The Swedish personal identity number: Possibilities and pitfalls in healthcare and medical research. *Eur J Epidemiol*. 2009;24(11):659-667. doi:10.1007/s10654-009-9350-y

4. Brooke HL, Talbäck M, Hörnblad J, et al. The Swedish cause of death register. *Eur J Epidemiol*. 2017;32(9):765-773. doi:10.1007/s10654-017-0316-1

5. Ludvigsson JF, Almqvist C, Bonamy AKE, et al. Registers of the Swedish total population and their use in medical research. *Eur J Epidemiol*. 2016;31(2):125-136. doi:10.1007/s10654-016-0117-y

6. Ludvigsson JF, Svedberg P, Olén O, Bruze G, Neovius M. The longitudinal integrated database for health insurance and labour market studies (LISA) and its use in medical research. *Eur J Epidemiol*. 2019;34(4):423-437. doi:10.1007/s10654-019-00511-8

7. Wettermark B, Hammar N, Fored CM, et al. The new Swedish prescribed drug register: opportunities for pharmacoepidemiological research and experience from the first six months. *Pharmacoepidemiol Drug Saf*. 2007;16(7):726-735. doi:10.1002/pds.1294

8. British Columbia Vital Statistics Agency [creator]. Vital Statistics Births: Population Data BC [publisher]; 2022. https://www.popdata.bc.ca/data/demographic/vs_births

9. British Columbia Vital Statistics Agency[creator]. Vital Statistics Deaths: Population Data BC [publisher]; 2022. Accessed October 5, 2022. https://www.popdata.bc.ca/data/demographic/vs_deaths

10. Canadian Institute for Health Information[creator]. Discharge Abstract Database (Hospital Separations) [Internet]. Population Data BC [publisher]; 2022. Accessed October 5, 2022. https://www.popdata.bc.ca/data/health/dad

11. British Columbia Ministry of Health [creator]. Medical Services Plan (MSP) Payment Information File. Population Data BC [publisher]; 2022. Accessed October 5, 2022. https://www.popdata.bc.ca/data/health/msp

12. British Columbia Ministry of Health [creator]. PharmaNet V2. Population Data BC [publisher]; 2022. Accessed October 5, 2022. https://www.popdata.bc.ca/data/health/pharmanet

13. Vanderweele TJ. *Explanation in Causal Inference: Methods for Mediation and Interaction*. Oxford University Press; 2015.

14. White IR, Royston P, Wood AM. Multiple imputation using chained equations: issues and guidance for practice. *Stat Med*. 2011;30(4):377-399. doi:10.1002/sim.4067

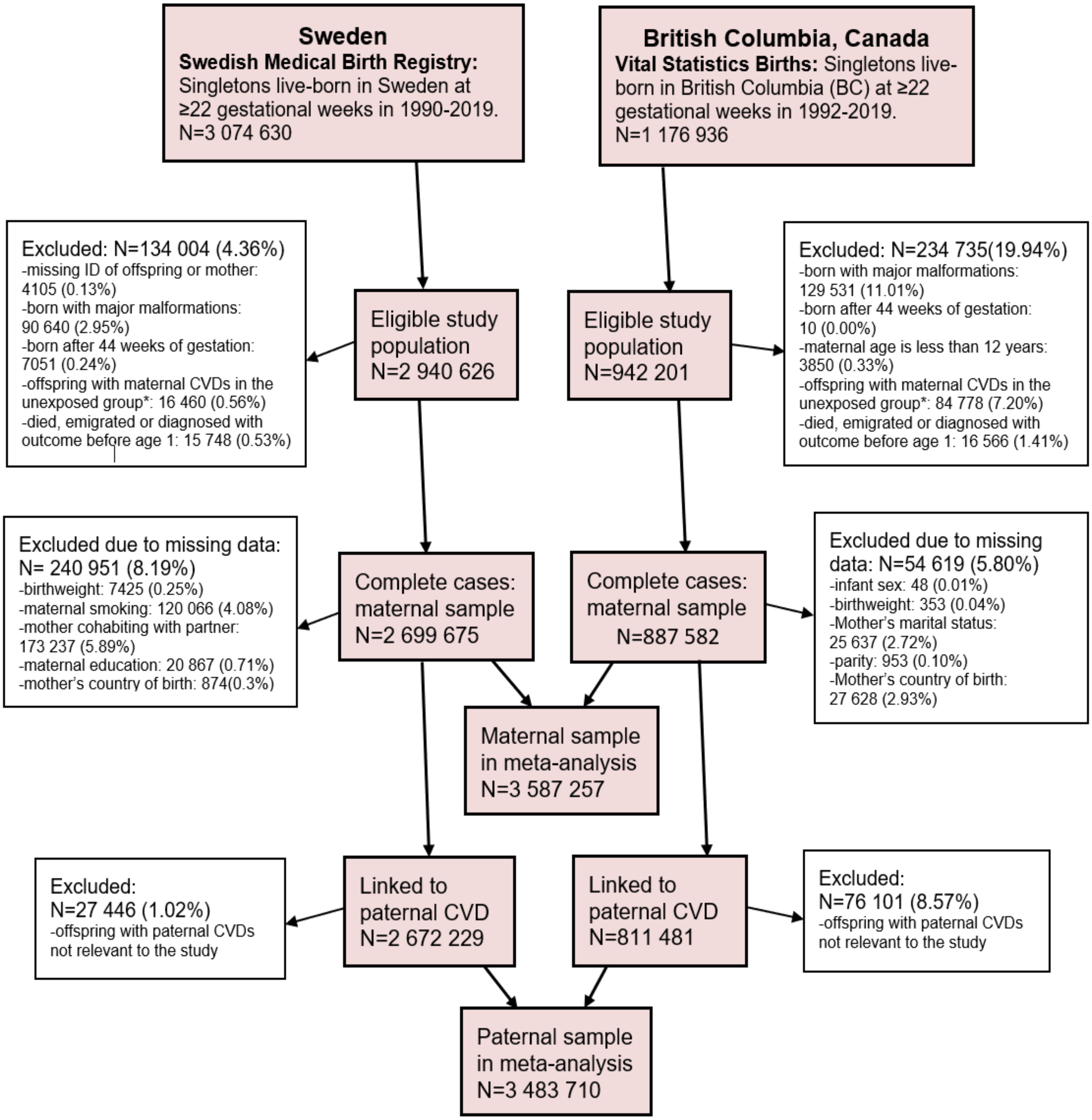

*Excluded from the unexposed group were the offspring of mothers diagnosed with any cardiovascular diseases, except those with the diagnoses for hypertensive diseases.

Supplementary Figure S1. Flow diagram of the study samples

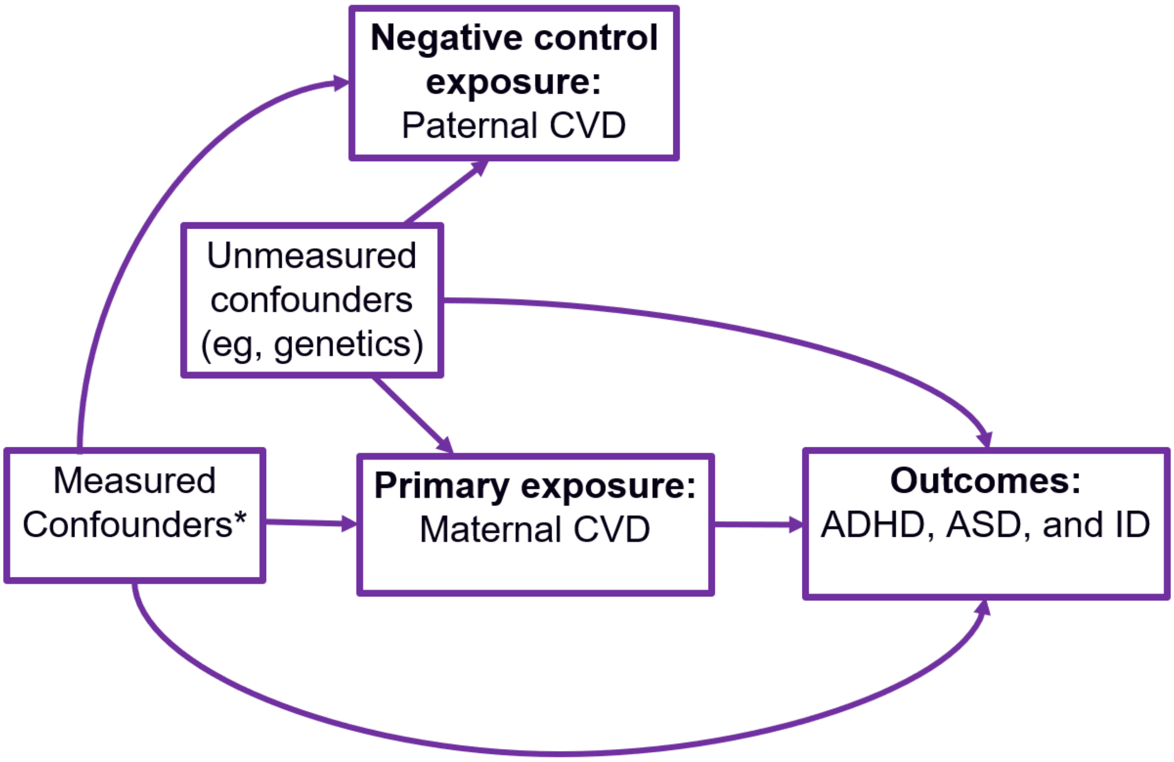

Supplementary Figure S2. A conceptual framework of the study

Note: ADHD, attention-deficit/hyperactivity disorder; ASD, autism spectrum disorder; CVD, cardiovascular disease; ID, intellectual disability.

*The measured confounders include mother's age at delivery, parity, education, country of birth, marital/cohabitation status, smoking during early pregnancy, pre-gestational diabetes, pre-gestational hypertension, and parental history of any neurodevelopmental or psychiatric disorders.

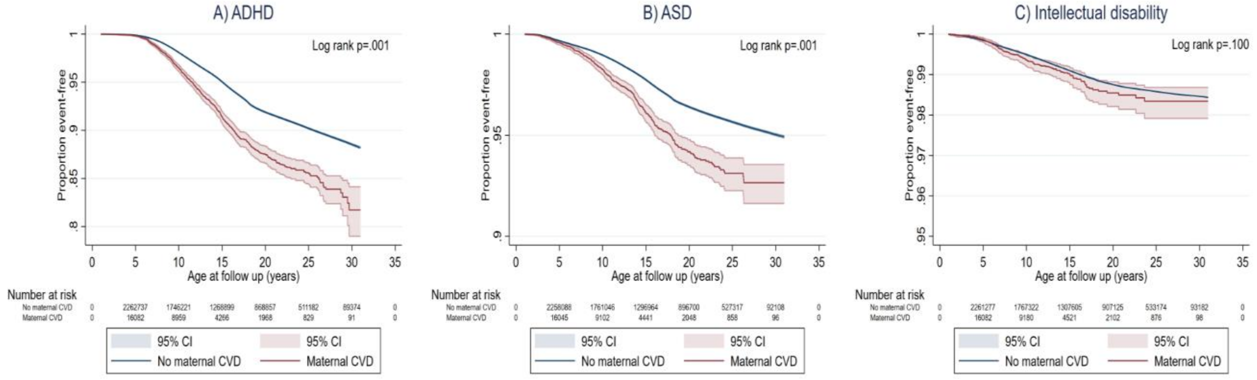

Supplementary Figure S3. Kaplan-Meier survival curves showing event-free survival probabilities by maternal cardiovascular disease status: singleton offspring live-born without major malformations in Sweden 1990 to 2019 (N=2 699 675).
Note: ADHD, attention-deficit/ hyperactivity disorder; ASD, autism spectrum disorder; CI, confidence interval.

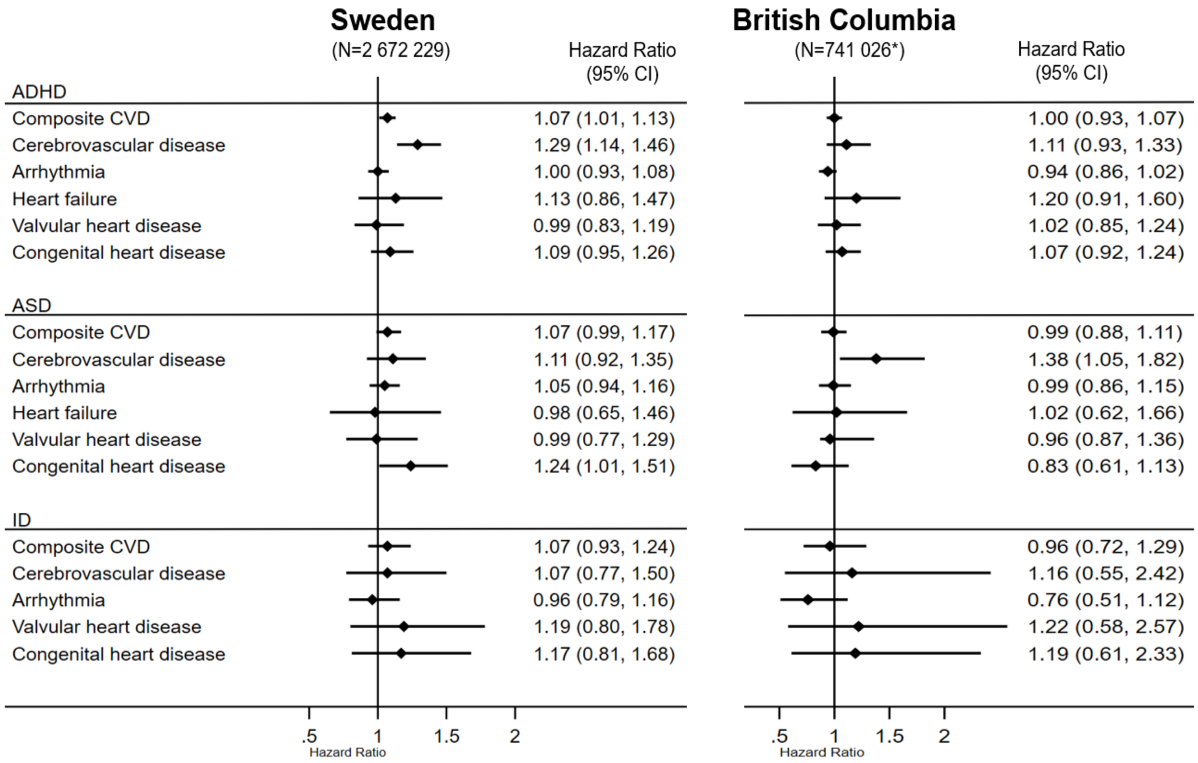

Supplementary Figure S4. Hazard ratios of neurodevelopmental disorders according to paternal cardiovascular disease, adjusted for paternal age at child's birth: singleton offspring live-born without major malformations in Sweden 1990 to 2019 (N=2 672 229) and British Columbia, Canada 1992 to 2019 (N= 741 026)

*Offspring with missing data on paternal age were excluded from BC data. The Swedish data had no missing on paternal age.

Note: ADHD, attention-deficit/hyperactivity disorder; ASD, autism spectrum disorder; CVD, cardiovascular disease; ID, intellectual disability. All hazard ratios were adjusted for child's age, sex, birthyear; paternal age at delivery; and mother's CVD, age at delivery, parity, education, country of birth, cohabitation with partner, smoking during pregnancy, pre-gestational diabetes, pre-gestational hypertension, and parental history of any neurodevelopmental or psychiatric disorders.

Supplementary Table S1. Register databases from which the study variables were derived

Major variables in the study	Register data sources	
	**Sweden**	**BC, Canada**
**Neonatal characteristics**		
Birth year	Medical Birth Register	Vital Statistics Births
Sex	Medical Birth Register	Vital Statistics Births
Preterm birth (<37 gestational weeks)	Medical Birth Register	Vital Statistics Births
Small for gestational age (<10th percentile)	Medical Birth Register	Vital Statistics Births
ADHD	National Patient Register<br>Prescribed Drug Register	Discharge Abstract Database<br>Medical Services Plan<br>PharmaNet
ASD	National Patient Register	Discharge Abstract Database<br>Medical Services Plan
ID	National Patient Register	Discharge Abstract Database<br>Medical Services Plan
**Maternal characteristics**		
Age at delivery	Medical Birth Register	Vital Statistics Births
Parity	Medical Birth Register	Vital Statistics Births
Marital/cohabitation status	Medical Birth Register	Vital Statistics Births
Region of birth	Total Population Register	Vital Statistics Births
Education	Education Register	Not available
Smoking during early pregnancy	Medical Birth Register	Not available
Pre-existing CVD	National Patient Register	Discharge Abstract Database<br>Medical Services Plan
Neurodevelopmental and psychiatric disorders	National Patient Register	Discharge Abstract Database<br>Medical Services Plan
Pre-gestational hypertension	National Patient Register<br>Medical Birth Register	Discharge Abstract Database<br>Medical Services Plan
Pre-pregnancy diabetes	National Patient Register<br>Medical Birth Register	Discharge Abstract Database<br>Medical Services Plan

Note: ADHD, attention-deficit/hyperactivity disorder; ASD, autism spectrum disorder; CVD, cardiovascular disease; ID, intellectual disability.

Supplementary Table S2. International Classification of Disease (ICD) codes used to define maternal diseases and offspring neurodevelopmental disorders

	ICD-9	ICD-10
**Maternal diseases**		
Cerebrovascular disease	430-438	I60-I69, G45
Arrhythmia	426-427	I44-I49
Heart failure	428	I50
Valvular heart disease (non-congenital)	394-397, 424	I05-I09, I34-I37
Congenital heart disease	745-747	Q20-Q26
Any psychiatric disorders	290-319	F00-F99
Neurodevelopmental disorders	299, 314, 317-319	F84, F70-F73, F78-F79, F90
Pre-gestational diabetes	250, 648A	E10-E14, O24.1-O24.3
Pre-gestational hypertension*	401-405, 642C, 642H	I10-I15, O10-O11
**Offspring neurodevelopmental disorders**		
ADHD[#]	314	F90
ASD	299	F84
ID	317-319	F70-F73, F78-F79

ADHD, attention-deficit/ dyperactivity disorder; ASD, autism spectrum disorder; ICD, international classification of disease; ID, intellectual disability.
*Pre-gestational hypertension was also self-reported in a checkbox at the first prenatal visit.
[#]The ADHD cases were additionally identified through dispensed drugs using the following Anatomical Therapeutic Chemical (ATC) codes, available since 2005 in Sweden and since 1996 in British Columbia: N06BA01 (amphetamine), N06BA02 (dexamfetamine), N06BA04 (methylphenidate), N06BA09 (atomoxetine), N06BA12 (lisdexamfetamine).

Supplementary Table S3. Sex-stratified incidence rates and hazard ratios of ADHD and ASD according to preterm birth: singleton offspring live-born without major malformations in Sweden 1990 to 2019 and in British Columbia, Canada 1992 to 2019

Sweden

Males (N=1 380 710)

	ADHD				ASD			
**Preterm birth**	No. of events	Rates[a]	Model 1 (95% CI)	Model 2 (95% CI)	No. of events	Rates[a]	Model 1 (95% CI)	Model 2 (95% CI)
No	90 061	4.9	1.00 (Ref.)	1.00 (Ref.)	40 083	2.1	1.00 (Ref.)	1.00 (Ref.)
Yes	5679	6.2	1.29 (1.26-1.33)	1.20 (1.17-1.23)	2656	2.9	1.36 (1.30-1.41)	1.25 (1.20-1.30)

Females (N=1 318 891)

**Preterm birth**								
No	55 612	3.1	1.00 (Ref.)	1.00 (Ref.)	20 356	1.1	1.00 (Ref.)	1.00 (Ref.)
Yes	3047	3.9	1.24 (1.19-1.28)	1.15 (1.11-1.19)	1341	1.7	1.49 (1.41-1.58)	1.38 (1.31-1.46)

British Columbia

Males (N=451 770)

**Preterm birth**								
No	51 287	9.8	1.00 (Ref.)	1.00 (Ref.)	13 660	2.4	1.00 (Ref.)	1.00 (Ref.)
Yes	3378	12.0	1.22 (1.18-1.27)	1.16 (1.12-1.20)	1012	3.3	1.32 (1.24-1.41)	1.25 (1.17-1.33)

Females (N=435 812)

**Preterm birth**								
No	22 395	4.2	1.00 (Ref.)	1.00 (Ref.)	4059	0.7	1.00 (Ref.)	1.00 (Ref.)
Yes	1263	5.2	1.24 (1.17-1.31)	1.16 (1.09-1.23)	287	1.1	1.51 (1.34-1.70)	1.39 (1.24-1.57)

Note: ADHD, attention-deficit/ hyperactivity disorder; ASD, autism spectrum disorder; CI, confidence interval; CVD, cardiovascular disease; HR, hazard ratio; ID, intellectual disability.
[a]Incidence rates per 1000 child-years.
Model 1 was adjusted for child's birthyear.
Model 2 was further adjusted for maternal CVD and mother's age at delivery, parity, education (Sweden only), region of birth, marital status/cohabitation with partner, smoking during early pregnancy (Sweden only), pre-gestational diabetes, pre-gestational hypertension, and parental history of any neurodevelopmental or psychiatric disorders.

Supplementary Table S4. Incidence rates and hazard ratios of neurodevelopmental disorders according to pre-existing maternal cardiovascular diseases, with versus without adjustment for maternal education and smoking: singleton offspring live-born without major malformations in Sweden 1990 to 2019 (N=2 699 675)

	**Outcomes**				
	**ADHD**				
**Exposure/s**	No. of events	Rates*	Model 1 HR (95% CI)	Model 2 HR (95% CI)	Model 3 HR (95% CI)
Composite maternal CVD					
No	153355	4.1	1.00 (Ref.)	1.00 (Ref.)	1.00 (Ref.)
Yes	1044	5.4	1.29 (1.21-1.37)	1.18 (1.11-1.26)	1.17 (1.10-1.25)
Subtypes of maternal CVD					
Cerebrovascular disease	204	6.0	1.40 (1.22-1.61)	1.30 (1.14-1.49)	1.21 (1.06-1.39)
Arrhythmia	565	5.2	1.24 (1.14-1.34)	1.14 (1.05-1.24)	1.16 (1.07-1.26)
Heart failure	26	6.4	2.04 (1.08-2.34)	1.47 (1.00-2.16)	1.44 (0.98-2.12)
Valvular heart disease	85	4.9	1.15 (0.93-1.43)	1.12 (0.91-1.39)	1.12 (0.91-1.39)
Congenital heart disease	239	5.4	1.35 (1.19-1.54)	1.18 (1.03-1.33)	1.17 (1.03-1.33)
	**ASD**				
Composite maternal CVD					
No	63974	1.7	1.00 (Ref.)	1.00 (Ref.)	1.00 (Ref.)
Yes	462	2.3	1.21 (1.10-1.32)	1.12 (1.02-1.23)	1.12 (1.02-1.23)
Subtypes of maternal CVD					
Cerebrovascular disease	99	2.9	1.54 (1.26-1.88)	1.37 (1.13-1.67)	1.35 (1.11-1.65)
Arrhythmia	242	2.2	1.11 (0.97-1.26)	1.04 (0.92-1.18)	1.04 (0.91-1.18)
Heart failure	18	4.4	2.12 (1.30-3.46)	2.05 (1.29-3.26)	1.92 (1.18-3.14)
Valvular heart disease	41	2.3	1.20 (0.88-1.65)	1.14 (0.84-1.55)	1.15 (0.84-1.57)
Congenital heart disease	108	2.4	1.24 (1.02-1.51)	1.16 (0.96-1.40)	1.15 (0.94-1.39)
	**ID**				
Composite maternal CVD					
No	22626	0.6	1.00 (Ref.)	1.00 (Ref.)	1.00 (Ref.)
Yes	132	0.7	1.06 (0.90-1.26)	1.00 (0.84-1.19)	0.99 (0.83-1.18)
Subtypes of maternal CVD					
Cerebrovascular disease	28	0.8	1.30 (0.90-1.88)	1.22 (0.84-1.76)	1.12 (0.77-1.62)
Arrhythmia	57	0.5	0.81 (0.63-1.05)	0.78 (0.60-1.01)	0.79 (0.61-1.03)
Heart failure	<5[!]	0.9	1.54 (0.58-4.11)	1.25 (0.47-3.33)	1.17 (0.44-3.10)
Valvular heart disease	19	1.1	1.73 (1.10-2.71)	1.53 (0.98-2.40)	1.49 (0.95-2.33)
Congenital heart disease	35	0.8	1.26 (0.90-1.75)	1.16 (0.83-1.61)	1.15 (0.83-1.61)

Note: ADHD, attention-deficit/hyperactivity disorder; ASD, autism spectrum disorder; CI, confidence interval; CVD, cardiovascular disease; HR, hazard ratio; ID, intellectual disability.

[#]Children with missing data on maternal body mass index were excluded.

*Incidence rates per 1000 child-years.

Model 1 was minimally adjusted for child's age, birth year and sex. Model 2 was further adjusted for mother's age at delivery, parity, region of birth, cohabitation with partner, pre-gestational diabetes, pre-gestational hypertension, and parental history of any neurodevelopmental or psychiatric disorders. Model 3 was additionally adjusted for maternal education and smoking during early pregnancy.

-Not estimated due to insufficient events

Supplementary Table S5. Incidence rates and hazard ratios of neurodevelopmental disorders according to pre-existing maternal cardiovascular diseases, adjusted for maternal early pregnancy body mass index: singleton offspring live-born without major malformations in Sweden 1992 to 2019 (N=2 311 311#).

**Exposure/s**	**Outcomes**				
	**ADHD**				
	No. of events	Rates*	Model 1 HR (95% CI)	Model 2 HR (95% CI)	Model 3 HR (95% CI)
Composite maternal CVD					
No	126 305	4.4	1.00 (Ref.)	1.00 (Ref.)	1.00 (Ref.)
Yes	945	5.4	1.28 (1.20-1.36)	1.16 (1.09-1.23)	1.17 (1.10-1.24)
Subtypes of maternal CVD					
Cerebrovascular disease	178	5.9	1.37 (1.18-1.58)	1.18 (1.01-1.36)	1.17 (1.01-1.36)
Arrhythmia	519	5.2	1.24 (1.14-1.35)	1.16 (1.06-1.27)	1.17 (1.07-1.28)
Heart failure	24	6.6	1.62 (1.09-2.42)	1.44 (0.97-2.15)	1.39 (0.93-2.07)
Valvular heart disease	76	4.8	1.12 (0.90-1.41)	1.09 (0.87-1.37)	1.10 (0.88-1.38)
Congenital heart disease	212	5.3	1.25 (1.08-1.45)	1.12 (0.98-1.28)	1.13 (0.99-1.30)
	**ASD**				
Composite maternal CVD					
No	53 337	1.8	1.00 (Ref.)	1.00 (Ref.)	1.00 (Ref.)
Yes	424	2.4	1.20 (1.09-1.32)	1.12 (1.01-1.23)	1.12 (1.02-1.24)
Subtypes of maternal CVD					
Cerebrovascular disease	84	2.8	1.41 (1.14-1.75)	1.25 (1.01-1.55)	1.25 (1.0-1.55)
Arrhythmia	228	2.3	1.13 (0.99-1.29)	1.07 (0.94-1.22)	1.08 (0.95-1.23)
Heart failure	17	4.6	2.32 (1.45-3.74)	2.08 (1.29-3.34)	2.00 (1.24-3.22)
Valvular heart disease	37	2.3	1.17 (0.85-1.61)	1.11 (0.81-1.53)	1.12 (0.81-1.54)
Congenital heart disease	97	2.4	1.21 (0.99-1.48)	1.012(0.92-1.37)	1.13 (0.93-1.38)
	**ID**				
Composite maternal CVD					
No	17 927	0.6	1.00 (Ref.)	1.00 (Ref.)	1.00 (Ref.)
Yes	121	0.7	1.07 (0.90-1.28)	1.00 (0.84-1.20)	1.01 (0.84-1.21)
Subtypes of maternal CVD					
Cerebrovascular disease	25	0.8	1.31 (0.88-1.94)	1.14 (0.77-1.69)	1.15 (0.77-1.70)
Arrhythmia	52	0.5	0.81 (0.62-1.06)	0.79 (0.60-1.04)	0.80 (0.61-1.05)
Heart failure	<5	1.1	-	-	-
Valvular heart disease	16	1.0	1.59 (0.97-2.59)	1.37 (0.84-2.24)	1.37 (0.84-2.25)
Congenital heart disease	32	0.8	1.25 (0.88-1.77)	1.15 (0.81-1.63)	1.16 (0.83-1.65)

Note: ADHD, attention-deficit/hyperactivity disorder; ASD, autism spectrum disorder; CI, confidence interval; CVD, cardiovascular disease; HR, hazard ratio; ID, intellectual disability.
[#]Children with missing data on maternal body mass index were excluded.
*Incidence rates per 1000 child-years.
Model 1 was minimally adjusted for child's age, birth year and sex. Model 2 was further adjusted for mother's age at delivery, parity, education, region of birth, cohabitation with partner, smoking during early pregnancy, pre-gestational diabetes, pre-gestational hypertension, and parental history of any neurodevelopmental or psychiatric disorders. Model 3 was additionally adjusted for maternal early pregnancy body mass index.
-Not estimated due to insufficient events

Supplementary Table S6. Incidence rates and hazard ratios of neurodevelopmental disorders according to pre-existing maternal cardiovascular diseases, adjusted for parental neighbourhood income quintiles: singleton offspring live-born without major malformations in British Columbia, Canada 1992 to 2019 (N= 863 440).

Exposure/s	Outcomes				
	**ADHD**				
	No. of events	Rates*	Model 1 HR (95% CI)	Model 2 HR (95% CI)	Model 3 HR (95% CI)
Composite maternal CVD					
No	75 859	7.2	1.00 (Ref.)	1.00 (Ref.)	1.00 (Ref.)
Yes	2037	9.6	1.28 (1.22-1.34)	1.14 (1.09-1.20)	1.14 (1.09-1.20)
Subtypes of maternal CVD					
Cerebrovascular disease	195	10.2	1.39 (1.20-1.62)	1.18 (1.01-1.37)	1.18 (1.02-1.37)
Arrhythmia	1270	9.6	1.25 (1.17-1.32)	1.12 (1.05-1.19)	1.12 (1.06-1.19)
Heart failure	68	11.3	1.48 (1.14-1.93)	1.30 (0.99-1.70)	1.30 (0.99-1.70)
Valvular heart disease	299	8.3	1.15 (1.02-1.30)	1.12 (0.99-1.26)	1.12 (0.99-1.26)
Congenital heart disease	389	10.8	1.44 (1.29-1.60)	1.19 (1.06-1.32)	1.19 (1.07-1.33)
	**ASD**				
Composite maternal CVD					
No	18 296	1.6	1.00 (Ref.)	1.00 (Ref.)	1.00 (Ref.)
Yes	611	2.7	1.27 (1.16-1.38)	1.16 (1.06-1.27)	1.17 (1.07-1.27)
Subtypes of maternal CVD					
Cerebrovascular disease	57	2.8	1.27 (0.94-1.72)	1.09 (0.81-1.47)	1.09 (0.81-1.48)
Arrhythmia	404	2.9	1.27 (1.14-1.41)	1.17 (1.05-1.30)	1.17 (1.06-1.30)
Heart failure	19	3.0	1.23 (0.75-2.00)	1.09 (0.67-1.77)	1.09 (0.67-1.77)
Valvular heart disease	73	1.9	1.21 (0.94-1.55)	1.14 (0.89-1.46)	1.14 (0.89-1.46)
Congenital heart disease	117	3.0	1.30 (1.07-1.59)	1.20 (0.98-1.46)	1.20 (0.98-1.46)
	**ID**				
Composite maternal CVD					
No	4005	0.3	1.00 (Ref.)	1.00 (Ref.)	1.00 (Ref.)
Yes	96	0.4	1.16 (0.95-1.42)	1.07 (0.88-1.31)	1.08 (0.88-1.32)
Subtypes of maternal CVD					
Cerebrovascular disease	13	0.6	1.77 (1.03-3.05)	1.56 (0.91-2.67)	1.56 (0.91-2.68)
Arrhythmia	62	0.4	1.19 (0.93-1.53)	1.11 (0.87-1.43)	1.12 (0.87-1.44)
Heart failure	<5	0.6	-	-	-
Valvular heart disease	9	0.2	0.65 (0.34-1.24)	0.65 (0.34-1.25)	0.65 (0.34-1.25)
Congenital heart disease	18	0.5	1.28 (0.80-2.02)	1.08 (0.68-1.71)	1.09 (0.69-1.73)

Note: ADHD, attention-deficit/hyperactivity disorder; ASD, autism spectrum disorder; CI, confidence interval; CVD, cardiovascular disease; HR, hazard ratio; ID, intellectual disability.
*Incidence rates per 1000 child-years.
Model 1 was minimally adjusted for child's age, birth year and sex. Model 2 was further adjusted for mother's age at delivery, parity, region of birth, marital status, pre-gestational diabetes, pre-gestational hypertension, and parental history of any neurodevelopmental or psychiatric disorders. Model 3 was additionally adjusted for parental neighbourhood income quintiles.
-Not estimated due to insufficient events

Supplementary Table S7. Multiple-imputation analysis of the associations between pre-existing maternal cardiovascular diseases and offspring's neurodevelopmental disorders: singleton offspring live-born without major malformations in Sweden 1990 to 2019 (N=2 940 626)

**Exposure/s**	**Outcomes**			
	**ADHD**			
	No. of events	Rates*	Model 1 HR (95% CI)	Model 2 HR (95% CI)
Composite maternal CVD				
No	168 725	4.1	1.00 (Ref.)	1.00 (Ref.)
Yes	1125	5.3	1.28 (1.21-1.36)	1.16 (1.09-1.23)
Subtypes of maternal CVD				
Cerebrovascular disease	222	6.0	1.40 (1.22-1.59)	1.20 (1.05-1.37)
Arrhythmia	603	5.1	1.22 (1.13-1.32)	1.14 (1.05-1.23)
Heart failure	28	6.3	1.55 (1.07-2.24)	1.39 (0.96-2.02)
Valvular heart disease	93	4.8	1.15 (0.94-1.41)	1.13 (0.92-1.38)
Congenital heart disease	269	5.6	1.39 (1.23-1.57)	1.18 (1.05-1.33)
	**ASD**			
Composite maternal CVD				
No	70 274	1.7	1.00 (Ref.)	1.00 (Ref.)
Yes	496	2.3	1.19 (1.09-1.30)	1.11 (1.01-1.21)
Subtypes of maternal CVD				
Cerebrovascular disease	106	2.8	1.46 (1.20-1.76)	1.28 (1.06-1.55)
Arrhythmia	256	2.2	1.07 (0.95-1.21)	1.01 (0.90-1.15)
Heart failure	20	4.4	2.08 (1.33-3.24)	1.90 (1.22-2.96)
Valvular heart disease	49	2.5	1.16 (0.87-1.55)	1.15 (0.86-1.53)
Congenital heart disease	118	2.4	1.18 (0.98-1.42)	1.11 (0.92-1.33)
	**ID**			
Composite maternal CVD				
No	25 315	0.6	1.00 (Ref.)	1.00 (Ref.)
Yes	150	0.7	1.10 (0.94-1.30)	1.03 (0.87-1.21)
Subtypes of maternal CVD				
Cerebrovascular disease	31	0.8	1.27 (0.89-1.81)	1.10 (0.77-1.56)
Arrhythmia	66	0.6	0.84 (0.66-1.08)	0.83 (0.65-1.06)
Heart failure	5	1.1	-	-
Valvular heart disease	21	1.1	1.59 (1.02-2.48)	1.41 (0.90-2.19)
Congenital heart disease	41	0.8	1.23 (0.90-1.69)	1.17 (0.85-1.60)

Note: ADHD, attention-deficit/hyperactivity disorder; ASD, autism spectrum disorder; CI, confidence interval; CVD, cardiovascular disease; HR, hazard ratio; ID, intellectual disability.
*Incidence rates per 1000 child-years.
Model 1 was minimally adjusted for child's age, birth year and sex. Model 2 was further adjusted for mother's age at delivery, parity, education, region of birth, cohabitation with partner, smoking during early pregnancy, pre-gestational diabetes, pre-gestational hypertension, and parental history of any neurodevelopmental or psychiatric disorders.
-Not estimated due to insufficient events

Supplementary Table S8. Sensitivity analysis restricted to later-born cohorts, concerning the associations between pre-existing maternal cardiovascular diseases and offspring's neurodevelopmental disorders: singleton offspring live-born without major malformations in Sweden 1997 to 2019 (N=2 0374 62).

Exposure/s	Outcomes			
	**ADHD**			
	No. of events	Rates*	Model 1 HR (95% CI)	Model 2 HR (95% CI)
Composite maternal CVD				
No	105 423	5.0	1.00 (Ref.)	1.00 (Ref.)
Yes	925	5.6	1.27 (1.19-1.36)	1.15 (1.08-1.23)
Subtypes of maternal CVD				
Cerebrovascular disease	171	6.3	1.37 (1.18-1.59)	1.18 (1.01-1.37)
Arrhythmia	506	5.3	1.22 (1.11-1.33)	1.13 (1.04-1.24)
Heart failure	23	6.6	1.57 (1.04-2.36)	1.38 (0.92-2.08)
Valvular heart disease	72	4.7	1.07 (0.85-1.35)	1.05 (0.83-1.32)
Congenital heart disease	215	5.7	1.35 (1.18-1.55)	1.16 (1.01-1.33)
	**ASD**			
Composite maternal CVD				
No	45 756	2.1	1.00 (Ref.)	1.00 (Ref.)
Yes	412	2.5	1.17 (1.06-1.29)	1.09 (0.99-1.20)
Subtypes of maternal CVD				
Cerebrovascular disease	83	3.0	1.37 (1.15-1.78)	1.27 (1.02-1.57)
Arrhythmia	222	2.3	1.10 (0.96-1.25)	1.04 (0.91-1.19)
Heart failure	17	4.8	2.31 (1.44-3.72)	2.07 (1.29-3.34)
Valvular heart disease	36	2.3	1.13 (0.82-1.57)	1.09 (0.78-1.50)
Congenital heart disease	94	2.5	1.18 (0.97-1.45)	1.10 (0.90-1.34)
	**ID**			
Composite maternal CVD				
No	13 740	0.6	1.00 (Ref.)	1.00 (Ref.)
Yes	114	0.7	1.05 (0.88-1.27)	1.00 (0.83-1.20)
Subtypes of maternal CVD				
Cerebrovascular disease	20	0.7	1.13 (0.73-1.76)	1.00 (0.65-1.55)
Arrhythmia	50	0.5	0.80 (0.61-1.06)	0.79 (0.60-1.05)
Heart failure	<5	1.1	-	-
Valvular heart disease	16	1.0	1.64 (1.01-2.68)	1.43 (0.88-2.34)
Congenital heart disease	33	0.9	1.35 (0.96-1.90)	1.26 (0.90-1.78)

Note: ADHD, attention-deficit/hyperactivity disorder; ASD, autism spectrum disorder; CI, confidence interval; CVD, cardiovascular disease; HR, hazard ratio; ID, intellectual disability.
*Incidence rates per 1000 child-years.
Model 1 was minimally adjusted for child's age, birth year and sex. Model 2 was further adjusted for mother's age at delivery, parity, education, region of birth, cohabitation with partner, smoking during early pregnancy, pre-gestational diabetes, pre-gestational hypertension, and parental history of any neurodevelopmental or psychiatric disorders.
-Not estimated due to insufficient events

Supplementary Table S9. Sensitivity analysis restricted to later-born cohorts, concerning the associations between pre-existing maternal cardiovascular diseases and offspring's neurodevelopmental disorders: singleton offspring live-born without major malformations in British Columbia, Canada 2001 to 2019 (N= 589 933).

Exposure/s	Outcomes			
	**ADHD**			
	No. of events	Rates*	Model 1 HR (95% CI)	Model 2 HR (95% CI)
Composite maternal CVD				
No	40 389	8.2	1.00 (Ref.)	1.00 (Ref.)
Yes	1430	10.5	1.31 (1.24-1.39)	1.14 (1.08-1.21)
Subtypes of maternal CVD				
Cerebrovascular disease	136	11.0	1.40 (1.17-1.68)	1.18 (0.99-1.42)
Arrhythmia	926	10.2	1.26 (1.18-1.35)	1.12 (1.05-1.20)
Heart failure	54	12.1	1.57 (1.18-2.09)	1.31 (0.98-1.77)
Valvular heart disease	178	11.3	1.35 (1.16-1.58)	1.27 (1.08-1.48)
Congenital heart disease	280	11.4	1.44 (1.26-1.64)	1.13 (0.99-1.28)
	**ASD**			
Composite maternal CVD				
No	13 596	2.7	1.00 (Ref.)	1.00 (Ref.)
Yes	527	3.7	1.29 (1.17-1.41)	1.19 (1.08-1.31)
Subtypes of maternal CVD				
Cerebrovascular disease	54	4.2	1.41 (1.03-1.92)	1.22 (0.89-1.66)
Arrhythmia	351	3.7	1.27 (1.14-1.42)	1.18 (1.06-1.32)
Heart failure	17	3.6	1.23 (0.73-2.08)	1.09 (0.64-1.84)
Valvular heart disease	55	3.3	1.29 (0.96-1.73)	1.20 (0.89-1.61)
Congenital heart disease	102	4.0	1.30 (1.05-1.61)	1.19 (0.96-1.48)
	**ID**			
Composite maternal CVD				
No	1959	0.4	1.00 (Ref.)	1.00 (Ref.)
Yes	70	0.5	1.31 (1.03-1.66)	1.21 (0.95-1.53)
Subtypes of maternal CVD				
Cerebrovascular disease	11	0.8	2.29 (1.27-4.14)	2.00 (1.11-3.59)
Arrhythmia	47	0.5	1.31 (0.98-1.75)	1.25 (0.94-1.68)
Heart failure	<5	-	-	-
Valvular heart disease	5	0.3	0.76 (0.32-1.83)	0.76 (0.31-1.82)
Congenital heart disease	12	0.5	1.25 (0.71-2.21)	1.02 (0.57-1.79)

Note: ADHD, attention-deficit/hyperactivity disorder; ASD, autism spectrum disorder; CI, confidence interval; CVD, cardiovascular disease; HR, hazard ratio; ID, intellectual disability.
*Incidence rates per 1000 child-years.
Model 1 was minimally adjusted for child's age, birth year and sex. Model 2 was further adjusted for mother's age at delivery, parity, region of birth, marital status, pre-gestational diabetes, pre-gestational hypertension, and parental history of any neurodevelopmental or psychiatric disorders.
-Not estimated due to insufficient events

Supplementary Table S10. Incidence rates and hazard ratios of autism spectrum disorder without intellectual disability according to pre-existing maternal cardiovascular diseases: singleton offspring live-born without major malformations in Sweden 1990 to 2019 (N=2 672 229) and British Columbia, Canada 1992 to 2019 (N= 887 582).

Exposure/s	No. of events	Rates*	Model 1 HR (95% CI)	Model 2 HR (95% CI)
	**Sweden**			
Composite maternal CVD				
No	54 907	1.4	1.00 (Ref.)	1.00 (Ref.)
Yes	402	2.0	1.23 (1.11-1.36)	1.14 (1.03-1.25)
Subtypes of maternal CVD				
Cerebrovascular disease	86	2.5	1.53 (1.24-1.89)	1.34 (1.09-1.66)
Arrhythmia	215	1.9	1.16 (1.01-1.33)	1.08 (0.95-1.24)
Heart failure	14	3.4	2.06 (1.22-3.48)	1.90 (1.12-3.20)
Valvular heart disease	32	1.8	1.10 (0.78-1.55)	1.06 (0.75-1.50)
Congenital heart disease	92	2.1	1.26 (1.02-1.54)	1.16 (0.94-1.42)
	**British Columbia, Canada**			
Composite maternal CVD				
No	16 965	1.5	1.00 (Ref.)	1.00 (Ref.)
Yes	574	2.5	1.27 (1.17-1.39)	1.16 (1.06-1.27)
Subtypes of maternal CVD				
Cerebrovascular disease	52	2.5	1.25 (0.92-1.70)	1.06 (0.78-1.45)
Arrhythmia	380	2.7	1.28 (1.15-1.42)	1.17 (1.05-1.30)
Heart failure	17	2.6	1.15 (0.70-1.90)	1.02 (0.62-1.67)
Valvular heart disease	70	1.8	1.27 (0.98-1.64)	1.18 (0.92-1.53)
Congenital heart disease	112	2.8	1.32 (1.08-1.62)	1.21 (0.99-1.49)

Note: CI, confidence interval; CVD, cardiovascular disease; HR, hazard ratio.
*Incidence rates per 1000 child-years.
Model 1 was minimally adjusted for child's age, birth year and sex. Model 2 was further adjusted for mother's age at delivery, parity, education (Sweden only), region of birth, marital status/cohabitation with partner, smoking during early pregnancy (Sweden only), pre-gestational diabetes, pre-gestational hypertension, and parental history of any neurodevelopmental or psychiatric disorders.
